# Personally meaningful life events from adolescence to young adulthood: a longitudinal natural language processing analysis

**DOI:** 10.1111/jcpp.70169

**Published:** 2026-06-22

**Authors:** David Bürgin, Christina Haag, Lynn Alison Büeler, Laura Bechtiger, Clarissa Janousch, Elena Feldmann, Denis Ribeaud, Manuel Eisner, Viktor von Wyl, Lilly Shanahan

**Affiliations:** ^1^ Jacobs Center for Productive Youth Development University of Zurich Zurich Switzerland; ^2^ Child and Adolescent Psychiatric Research Department University Psychiatric Hospitals, University of Basel Basel Switzerland; ^3^ Institute for Implementation Science in Health Care University of Zurich Zurich Switzerland; ^4^ Epidemiology, Biostatistics and Prevention Institute University of Zurich Zurich Switzerland; ^5^ MRC Cognition and Brain Sciences Unit, University of Cambridge Cambridge UK; ^6^ Experimental and Clinical Pharmacopsychology, Department of Adult Psychiatry and Psychotherapy University Hospital of Psychiatry Zurich, University of Zurich Switzerland; ^7^ Institute of Criminology University of Cambridge Cambridge UK; ^8^ Department of Psychology University of Zurich Zurich Switzerland

**Keywords:** Life events, adolescence, natural language processing, topic modeling, lived experience

## Abstract

**Background:**

Large‐scale population‐based studies of risk and protective factors for youth mental health rarely assess youths' first‐hand experiences in their own words. This longitudinal study analyzed young people's self‐reported most important life events and examined how the key topics changed from midadolescence to young adulthood and are associated with internalizing symptoms.

**Methods:**

*N* = 1,442 participants from an urban, multiethnic cohort reported their most important life events at ages 15, 17, 20, and 24 in open‐text segments. Themes in *N* = 5,670 descriptions were analyzed using topic modeling with the Python library BERTopic.

**Results:**

Most reported events were positive in valence (83.1%), and were often ‘normative’, commonly occurring experiences spanning diverse life domains (education and career development, social relationships, leisure activities and successes, mental health and well‐being, and other life transitions and independence). Event topics displayed systematic developmental shifts from midadolescence to adulthood. Higher internalizing symptoms were associated with a greater likelihood of reporting interpersonal and stressful events rather than positive or achievement‐related ones.

**Conclusions:**

Longitudinal population‐based research can draw on open‐text data and employ NLP techniques to assess young people's lived experiences. The predominance of positive events and their associations with internalizing symptoms suggest that an increased focus on promoting beneficial experiences, next to reducing stressful events, may promote youth mental health.

## Introduction

Youth mental health research has been criticized for repeatedly analyzing the same sets of risk and protective factors, without sufficiently explaining recent declines in youth mental health or providing insights to reverse this trend (Armitage, Collishaw, & Sellers, 2024; Franklin et al., 2017; McGorry et al., 2024; Mojtabai & Olfson, 2020; Sonuga‐Barke, 2023). Asking young people directly about what is important to them and analyzing bottom‐up data with inductive designs may help generate new insights. Prior work on youth adjustment during the COVID‐19 pandemic (Lossio‐Ventura, Lee, Hancock, Linos, & Linos, 2021; Shanahan et al., 2023) and on drivers of mental distress and social withdrawal (Beccaria, Scavarda, Roggero, & Rabaglietti, 2022; Cecchin, da Costa, Pacheco, de Valencia, & Murta, 2024) illustrates how such approaches can highlight important but easily overlooked risk and protective factors (Shanahan et al., 2023; Sonuga‐Barke, 2024). Embracing such inductive strategies contributes to methodologically pluralistic translational research cycles and might help to advance youth mental health science (Sonuga‐Barke, 2023). However, such work is rare in large‐scale population‐based developmental research.

Adolescence and young adulthood are key formative periods of psychological and emotional development and major social transitions into roles and responsibilities, such as completing school, entering the workforce, or moving out of the parental home (Arnett, 2023; Sawyer, Azzopardi, Wickremarathne, & Patton, 2018). In particular, midadolescence to young adulthood are important phases in which educational and occupational trajectories begin to diverge, setting the course for adult life (Blokker et al., 2023). Moreover, research has highlighted that many positive life events peak in young adulthood and are commonly related to the timing of major developmental or transitional events (e.g., childbirth, marriage, or college graduation) (Berntsen, Rubin, & Siegler, 2011; Vuolo et al., 2014). Better understanding which life events are deemed most important by young people in this phase of life is essential for designing meaningful support.

Quantitative measures have been prioritized over text data in mental health research in recent decades, due in part to the considerable time required to systematically analyze text. However, the increasing accessibility of natural language processing (NLP) and large language models (LLMs) have revolutionized textual analysis by enabling the efficient extraction of themes and emotions from large‐scale text data (Evans, 2014; Hussain, Binz, Mata, & Wulff, 2024; Rohrer, Brummer, Schmukle, Goebel, & Wagner, 2017; Wolf et al., 2019). Advances in NLP enable nuanced analysis of individual narratives as a complement to traditional quantitative metrics. For example, during the COVID‐19 pandemic, a study of nearly 5,000 people used computational NLP‐based topic modeling to elucidate ‘silver linings’ (i.e., positive aspects of the pandemic), such as a sense of community, closeness, gratitude, and a belief in positive social change (Lossio‐Ventura et al., 2021). In multiple sclerosis (MS) research, NLP‐based topic modeling analyses have revealed patients' own subjective theories of MS‐incidence (e.g., stressors or mental health issues), and experiences of living with MS (e.g., the effects of MS on one's work and romantic partnership) (Haag et al., 2023, 2024). In young adults, NLP approaches have also recently highlighted diverse topics related to positive autobiographical memories (e.g., of close others or of being in nature and participating in outdoor activities) increasing well‐being (Haag, Philipp, & Kleim, 2023). These examples showcase that asking questions in an open‐text format elucidates important but easily overlooked subjective contributors to distress and well‐being. Despite the computational advances in investigating unstructured ‘qualitative’ text data, the full potential of integrating textual analyses into developmental and population‐based mental health research remains largely untapped.

Major life events are commonly defined as environmental changes within definable time periods associated with at least some level of behavioral and adaptive demands. These events can lead to unpleasantness, distress, and psychological threats, but they may also increase fulfillment and well‐being (Harkness & Monroe, 2016; Monroe & Slavich, 2019; Yarrington et al., 2023). Negative and stressful life events are among the most studied risk factors for mental and physical health problems (Cohen, Murphy, & Prather, 2019; Copeland, Keen, Tong, & Shanahan, 2024; Epel et al., 2018), particularly those occurring in childhood (e.g., neglect, violence exposure, and parental loss) (Hughes et al., 2017; LeMoult et al., 2020). Most stressful life events do not occur randomly but are observed along social gradients (e.g., sex/gender, socioeconomic status [SES], or immigration status) (Cohen et al., 2019; Sangalang et al., 2019). Although research has focused primarily on stressful life events, positive and promotive youth experiences have been proposed as an important, yet understudied, avenue for supporting mental health development (Berntsen et al., 2011; Han, Dieujuste, Doom, & Narayan, 2023; Morris, Hays‐Grudo, Zapata, Treat, & Kerr, 2021; Narayan, Merrick, Lane, & Larson, 2023).

Both stressful negative and positive life events lack a unified conceptualization, and no single, universally accepted measurement standard exists. While several widely used checklists for negative life events (e.g., adolescent and trauma‐focused life event inventories) are available, there is still considerable heterogeneity in item content, time frames and scoring approaches across studies (Cohen et al., 2019). Life events are typically assessed using self‐report checklists on a range of preselected events across life areas or resource‐intensive (semi‐)structured interviews (Monroe & Slavich, 2019). What is considered a ‘major life event’ is commonly predefined by investigators and their chosen scales or interview protocols, rarely by participants themselves (Monroe & Slavich, 2019). Moreover, short and feasible quantitative self‐report measures may not be developmentally sensitive or cover youths' lived experiences across developmental stages. In turn, resource‐intensive qualitative interviews have been difficult to implement at larger scales.

This study investigated open‐ended text data on young people's most important life events using NLP‐based topic modeling, drawing on data from an urban multiethnic cohort followed from midadolescence (age 15) into young adulthood (age 24). Using an inductive analytical approach, we identified key life event topics, classified their emotional valence, and examined how these topics changed across these developmental periods. We also analyzed how life event topics varied across sex, SES, and migration background. Finally, we tested internalizing symptoms as a cross‐sectional correlate of life event topics and valence.

## Methods

### Study design and procedures

Data came from the Zurich Project on Social Development from Childhood to Adulthood (z‐proso), a large, prospective‐longitudinal community study in Zurich, Switzerland. In 2004, the study used a cluster‐randomized sampling approach to select 56 primary schools in Zurich and recruit an initial target sample of 1,675 first graders (age seven) (Ribeaud, Murray, Shanahan, Shanahan, & Eisner, 2022). The design aimed to reproduce the composition of the children entering the city's public schools, while slightly overrepresenting low‐SES districts for the purposes of violence prevention research (Ribeaud et al., 2022). Approximately three‐quarters of children had at least one parent born outside of Switzerland (>90 countries), while about 90% of the participants themselves were born in Switzerland (Ribeaud et al., 2022). Nine assessment waves were conducted when participants were 7, 8, 9, 11, 13, 15, 17, 20, and 24 years old. A detailed study description can be found elsewhere (Ribeaud et al., 2022).

The current study used data from the last four waves of data collection, spanning the ages of 15–24 years (last assessment in 2022) (z‐proso Project Team, 2024). The assessments at ages 15 and 17 were conducted in schools, using standardized paper–pencil questionnaires. At ages 20 and 24, participants completed computer‐assisted questionnaires in a university laboratory in Zurich or participated remotely (*n*_Age 20_ = 37 and *n*_Age 40_ = 173) (Ribeaud et al., 2022). At age 15, the mean assessment date was March 29, 2013 (range: March 11, 2013 to July 4, 2013). For the age 17 assessment, the mean date was April 1, 2015 (range: March 7, 2015 to August 11, 2015). At age 20, assessments were conducted with a mean date of May 23, 2018 (range: April 17, 2018 to September 23, 2018). The final assessment at age 24 had a mean date of April 9, 2022 (range: February 28, 2022 to August 4, 2022). Participants received monetary compensation, which increased from ~30 USD at age 13 to ~150 USD at age 24.

Overall, 1,442 participants contributed text data on the main measure of interest on at least one occasion. At age 15, 1,299 of 1,446 participants provided a textual response (89.8%); at age 17, 1,153 of 1,306 participants (88.3%); at age 20, 1,135 of 1,180 participants (96.2%); and at age 24, 1,099 of 1,159 participants (94.8%). At age 15, the entire initial z‐proso target sample (*N* = 1,675) was recontacted, as young people were now able to provide their own informed consent. Because of this rerecruitment, 86.4% (*n* = 1,446) of the target sample took part at age 15. A comparison of the rerecruited group with the original target cohort showed almost no differences in participation by neighborhood disadvantage or migration background, indicating that the recontact procedures largely removed any recruitment or attrition bias (Eisner, Murray, Eisner, & Ribeaud, 2018; Ribeaud et al., 2022). As is the case with many longitudinal studies, attrition at later waves was generally slightly higher among males, youth with a migration background, and youth from low‐SES families (Eisner et al., 2018). This study was approved by the Ethics Committee of the Faculty of Arts and Social Sciences at the University of Zurich.

### Measures

#### Major life events

From age 15, the z‐proso assessments began with an open‐ended question about participants' most important life event since the last survey. Participants were asked, ‘If you think back over the last [two/three/four] years: What was, for you personally, the most important event in your life?’ Participants responded in an open text‐based format, writing down key words or short phrases. This item was included as a simple opener and an easy start to the survey, designed to convey genuine interest in the lives of the participants.

#### Sociodemographics

Child sex assigned at birth was coded from official school records (0 = male, 1 = female). Family SES was assessed using the International Socio‐Economic Index of Occupational Status based on parental occupation reported by adolescents (Ganzeboom, De Graaf, & Treiman, 1992). This score captures occupation‐specific income and required educational level (ranging from 16 [e.g., unskilled worker] to 90 [e.g., judge]). A composite score was created based on information gathered when participants were 11, 13, and 15 to maximize information. Migration background was assessed using parental country of birth reported by study participants (0 = no migration background [at least one parent born in Switzerland], 1 = migration background [both parents born abroad]).

#### Internalizing symptoms

At all measurement timepoints, symptoms of anxiety and depression were assessed using eight items from the youth/adult version of the Social Behavior Questionnaire addressing depressive and anxiety symptoms in the past month (Murray, Eisner, & Ribeaud, 2019; Murray, Obsuth, Eisner, & Ribeaud, 2019). Items were answered on a 5‐point scale labeled ‘never’, ‘rarely’, ‘sometimes’, ‘often’, or ‘very often’. The eight items focused on feeling sad without reason, unhappy, or alone (for depression), as well as on crying, being fearful, or worrying (for anxiety). The measurement properties of the SBQ have been widely tested, including in this sample (Murray, Eisner, & Ribeaud, 2019; Murray, Obsuth, et al., 2019). The scale was standardized before being used in the analyses.

### Data analysis

All NLP analyses were implemented in Python version 3.11.11 using the Jupyter Notebook environment version 7.3.2. Below, we provide information about how we proceeded with the NLP analysis. For reproducibility purposes, we provide additional information in Appendices S1 and S2; furthermore, the executed Jupyter notebook scripts for all NLP‐based analyses are available as Appendices S3 and S4.

#### Text data preprocessing

Before data analyses, text data (*n* = 5,550) was manually checked for spelling errors, punctuation, and language inconsistencies. Details for this process are further described in Appendix S1. A total of 35 text segments were excluded because they did not reference any specific event (e.g., ‘There were many events, several were important,’ or ‘A lot happened, but nothing specific comes to mind’), resulting in 5,515 text segments.

#### Topic modeling

Topic modeling was conducted with the BERTopic library in Python (Grootendorst, 2022). The overall methodological procedure is visualized in Figure 1.

**Figure 1:**
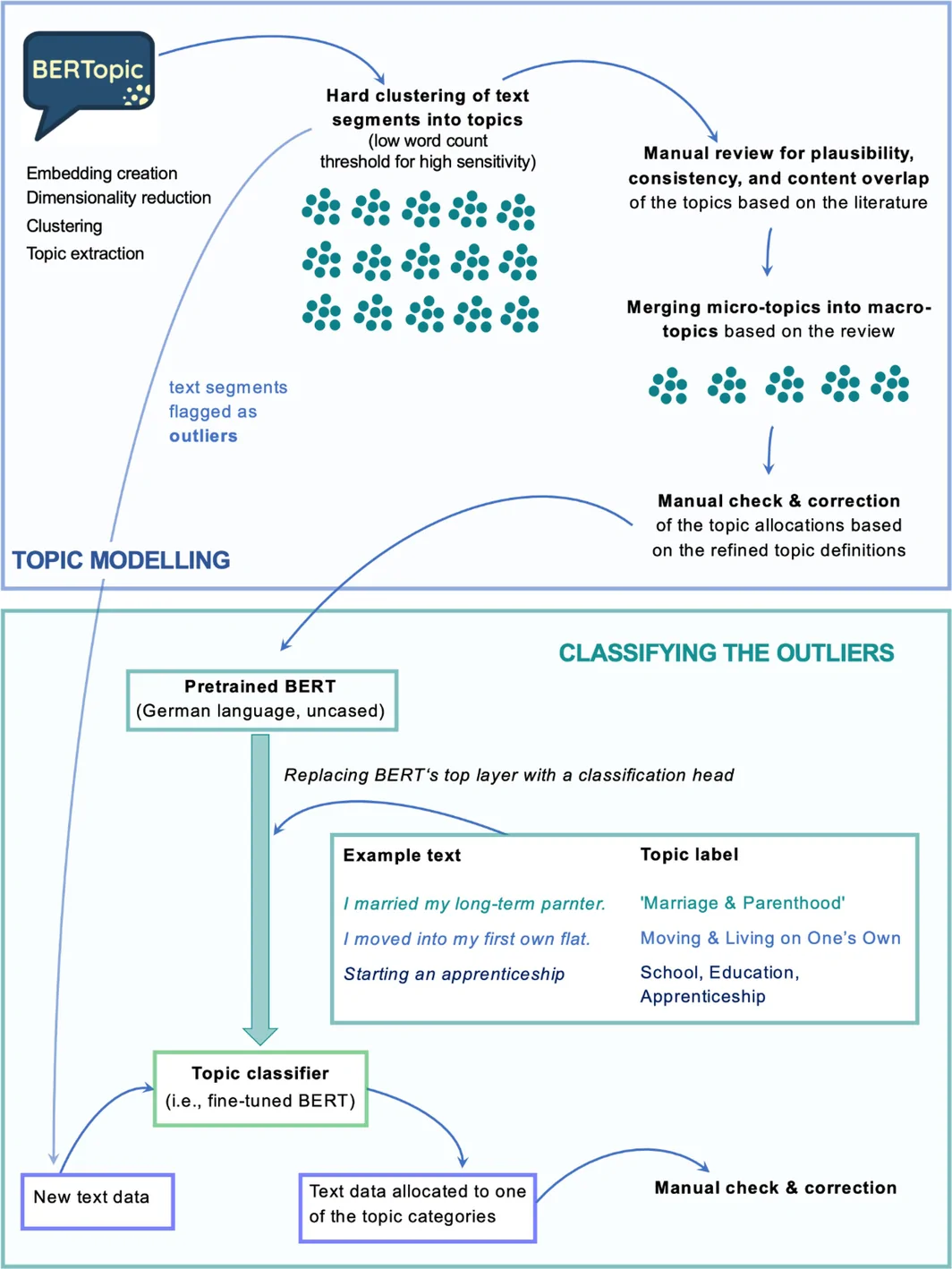
*Upper panel*: Topic Modeling using BERTopic. The figure outlines analysis steps of topic modeling implemented in the present study, consisting of data‐driven parts and manual reviews/checks. *Lower panel*: Allocation of the text segments flagged as outliers during initial topic modeling to the 12 topic categories using a fine‐tuned large language model

#### Topic formation process

##### Creating the embeddings

Text data were first transformed into numerical representations using the paraphrase‐multilingual‐MiniLM‐L12‐v2 sentence transformer model (Grootendorst, 2022; Reimers, 2019), which is suitable for the German language.

##### Dimensionality reduction

To reduce the complexity of the embeddings, we applied UMAP, a dimensionality reduction algorithm available in BERTopic (Grootendorst, 2022).

##### Hard clustering

Hard clustering of the reduced embeddings into topics was performed using the HDBSCAN algorithm built into BERTopic (Grootendorst, 2022). HDBSCAN was chosen because it automatically determines the number of topics, unlike k‐means, which requires a predefined number. Additionally, HDBSCAN handles irregularly shaped clusters, making it well‐suited for real‐world data where topics are rarely spherical or evenly distributed. This makes it particularly effective for overlapping and unbalanced topic distributions in BERTopic. During hard clustering, each text segment is allocated to a specific topic or marked as an outlier. The minimum number of text segments required to form a topic needs to be specified by the researcher. To ensure that rare microtopics mentioned by only a few participants were not overlooked, we set this minimum threshold at 30 text segments (i.e., <0.01% the 5,515 text segments). This allowed for a sensitive approach while maintaining topic relevance.

##### Merging overlapping topics

Due to the low threshold set for the minimum number of text segments required to form a topic, a high number of topics (32 unique topics) were automatically generated. To refine the topic structure, the topic allocation of all text segments was manually reviewed by two authors (CH and LAB, initials used throughout for transparency). The review revealed overlapping content across several topics (for full details, see the executed Jupyter notebook script ‘Appendix S3’). In particular, 11 topics were found to relate to various aspects of education systems and qualifications (e.g., grammar school, school‐leaving certificate, vocational college). Following a discussion among CH, DB, and LAB, it was decided to merge these into a single overarching education topic, rather than retaining numerous narrowly defined microtopics on education. There was some minor overlap among other topics—for example, between ‘Family & Family‐Related Events’ and ‘Travelling, Vacation, Staying Abroad.’ After consolidation, the final topic structure consisted of 12 topics, with only moderate to minimal content overlap remaining.

##### Manual review and correction of the topic assignments to the text segments

A total of 4,569 text segments had been assigned to topic during initial topic modeling. Next, CH, DB, and LAB reviewed and discussed the 12 resulting topics in terms of their content, evaluating them for plausibility, consistency, and alignment with the scientific literature. Based on these discussions, the core content of each topic was defined (see Table S1 for the summaries). Then, LAB and CH manually reviewed all topic assignments for the text segments and corrected any assignments that clearly deviated from the defined topic content. Text segments containing content relevant to more than one distinct topic were manually split, which occurred in 137 cases. This resulted in a total of 4,706 text segments that had been assigned a topic at this stage of the modeling process. Corrections were made in 23.93% of cases (including those that were manually split), a rate consistent with previous BERTopic research (Haag et al., 2024). Unclear cases were jointly reviewed by LAB and CH and resolved through discussion. Text segments classified as outliers were not considered at this point.

##### Handling ambiguous text segments excluded during initial topic modeling

Since BERTopic designates uncertain text segments as outliers rather than forcing them into topics, it typically results in a high number of outliers. In our case, 946 (20.10%) of all text segments were flagged as outliers during initial topic modeling, aligning with findings from previous research using BERTopic (Haag et al., 2024). As an additional step, these ambiguous segments can be assigned to the most relevant topics, a process referred to as ‘soft clustering’. This two‐step approach helps maintain topic precision and interpretability by preventing the dilution caused by forcing noisy data into predefined topics. Although BERTopic includes an internal procedure for soft clustering (Grootendorst, 2024a), we opted to train a classifier based on a large language model, as the internal method demonstrated limited performance in accurately assigning outliers.

#### Training an LLM‐based classifier to allocate outlier text segments across the 12 topic categories

For an overview of the analytic procedure, see Figure 1.

##### Step 1—ground truth data for model training

The manually reviewed text segments, along with their reviewed topic assignments from the initial topic modeling, served as the ground truth data for model training. The topic category represents the target variable that the large language model was trained to predict for new, unseen text data.

##### Step 2—subdividing the ground truth data into training and validation datasets

Following best practice when training a classification model, we divided our data into training (80%) and validation subsets (20%), stratifying for the 12 topic categories.

##### Step 3—model training

We trained the LLM‐based classifier to identify the outlined categories from text entries by replacing the top layer of the German BERT large language model (uncased) with a classification head [see for conceptual overview (Demszky et al., 2023)], a process also referred to as model fine‐tuning. For model training, we used the ‘Trainer’ class (The Hugging Face team (past and future) with the help of all our contributors, 2025) from the Transformers Python library (Wolf et al., 2019) and trained the model for 10 epochs.

##### Step 4—classifier evaluation

We focused on accuracy, F1 score, recall, and precision. Accuracy refers to the percentage of correct predictions out of the total number of predictions made by a classifier. Precision measures how many of the model's predictions for a class are correct, with high precision meaning fewer false positives. Recall measures how well the model identifies all correct cases for a class so that high recall means fewer false negatives. The F1 score balances precision and recall, ensuring the model minimizes both false positives and false negatives. For evaluation, we monitored metrics throughout training and selected the best‐performing model based on the F1 score (for full details, see the executed Jupyter notebook script ‘Appendix S3’; the best model was trained in epoch 5, F1 score = 0.885).

##### Step 5—Applying the classifier and manual review of the predicted classifications

We then applied the classifier to the text segments identified as outliers during the initial topic modeling. To validate model performance, all predictions were manually checked by LAB and CH. Since 18 text segments contained content relevant to more than one distinct topic, they were manually split, resulting in a total of 964 outlier text segments. Corrections were made to 13.2% of all outlier text segments, including those that were manually split.

#### Topic representation process

##### Lemmatization and noun extraction

The text data was tokenized using the CountVectorizer from the Scikit‐Learn library (Pedregosa et al., 2011), with all words lemmatized to their root forms (e.g. ‘friends’ to ‘friend’). As nouns provide the most relevant information for the study, lemmatized nouns were extracted from the text using the part‐of‐speech tagging functionality in the spaCy pipeline ‘de_core_news_sm’ (Altinok, 2021; spaCy, 2024).

##### Determining importance scores

To determine the importance of each word within a topic, BERTopic applies the statistical metric class‐based term frequency‐inverse document frequency (Grootendorst, 2022). Since the text segments were manually corrected and split where necessary, the total number of segments increased beyond what the original BERTopic model was trained on. Therefore, the most stable approach to generating topic representations was to use BERTopic's manual topic modeling option, which allows predefined categories – in this case 12 – to be converted directly into topic representations (for full details, see the executed Jupyter notebook script ‘Appendix S3’) (Grootendorst, 2024b).

##### Visualization of the importance scores

Using the Python WordCloud library (Mueller, 2020), we visualized the final topics, creating a separate graph for each topic, where word size reflects its importance score within a specific topic (see Figure 2). Building upon the 12 topics resulting from the data‐driven topic modeling approach, higher‐order categories were created by the research team based on topics with common and overlapping content to simplify readability and structure. This step was performed after we conducted a thorough review of the literature, discussed findings with multiple coauthors (LB, CJ, VvW, and LS), and presented preliminary findings to different groups of researchers.

**Figure 2:**
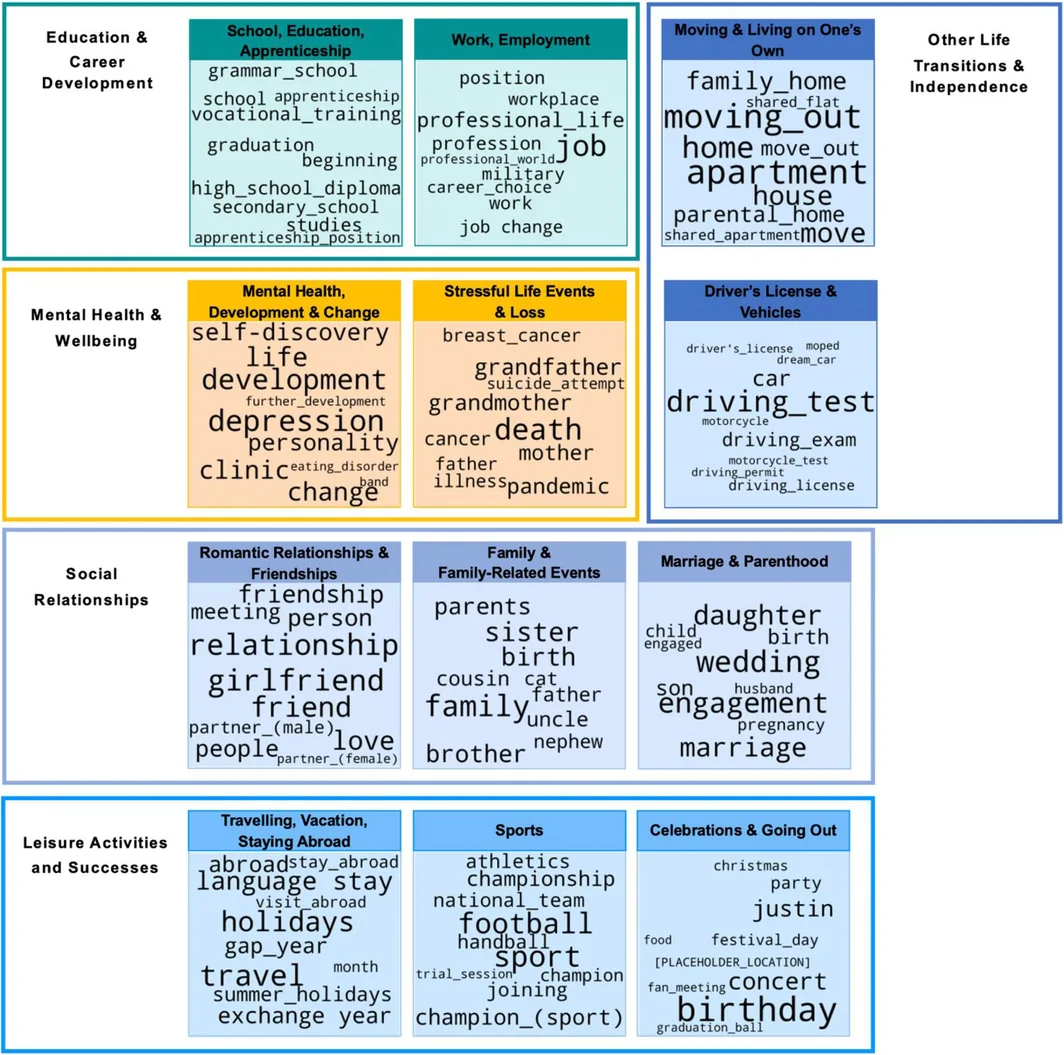
Results of the topic modeling analysis displaying the most important event in young people's lives in recent years, reported between ages 15 and 24, *N* = 5,670. The figure shows the 12 distinct topics identified by the topic modeling analysis using BERTopic. Text data within the word clouds were translated from German to English. The 12 topics have been grouped into five higher‐order categories based on thematic contents. The color scheme indicates a topic's classification into one of these five higher‐order categories: (1) education and career development (turquoise), (2) mental health and well‐being (yellow), (3) social relationships (mid/gray blue), (4) leisure activities and successes (sky blue), and (5) other life transitions and independence (dark blue). For readability, word sizes—reflecting their importance scores—are only comparable within each individual topic. The raw importance scores, which enable comparison across topics, are provided in Appendix S3 (see executed Jupyter notebook script ‘Appendix S3’).

#### Training an LLM‐based classifier for emotional valence

Furthermore, we trained an LLM‐based classifier to predict emotional valence of text segments. The analysis procedure is largely equivalent to the topic classifier described above; accordingly, this section is described in detail within the Supporting Information in ‘Appendix S2’ and ‘Figure S1’. Training an LLM‐based classifier for emotional valence and the executed Jupyter notebook script ‘Appendix S4’.

#### Characterization of life event topics

We performed descriptive analyses to describe topics occurrence and emotional valence across all text segments (*N* = 5,620, excl. 50 outlier text segments). To test sociodemographic predictors of life event topics for all text segments across all measurement timepoints, we used logistic regressions with cluster‐robust standard errors. The sample size for these models was *n* = 4,375 participants with unique text data contributions per wave and without missing sociodemographic data (6.6% of all *n* = 4,686 unique participants*age). In a second set of logistic regression models, we included the factor of age (ages 15, 17, 20, and 24) to test age‐related differences in topic frequencies. In a third set of models, we included internalizing symptoms as a predictor of life event topics, as well as for positive and negative life event valence. Requirements for these models were tested with the ‘check_models’ function in the performance package in R (Lüdecke, Ben‐Shachar, Patil, Waggoner, & Makowski, 2021). To visually display shifts in topics over the different ages, we used alluvial plots with the ‘ggalluvial’ package in R (Brunson & Read, 2023). We randomly selected one topic per measurement timepoint and individual if multiple topics were available. For these analyses, we used R (version 4.4.2, 2024‐10‐31) (R Core Team, 2024) through RStudio (version 2024.12.0.467) (Posit Team, 2024). We performed complete case analyses of all available data. Two‐sided significance testing was conducted, with corresponding alpha levels *< .05, **< .01, and ***< .001.

## Results

Overall, we investigated *N* = 5,670 distinct life event text segments (*n*_age 15_ = 1,444, *n*_age 17_ = 1,267, *n*_age 20_ = 1,448, *n*_age 24_ = 1,512). Individual participants (*N* = 1,442) reported a mean of 3.93 life events (SD = 1.65, range; Armitage et al., 2024; Arnett, 2023; Beccaria et al., 2022; Cecchin et al., 2024; Franklin et al., 2017; Lossio‐Ventura et al., 2021; McGorry et al., 2024; Mojtabai & Olfson, 2020; Sawyer et al., 2018; Shanahan et al., 2023; Sonuga‐Barke, 2023; Sonuga‐Barke, 2024) across all four timepoints. Fifty text entries (0.9%) were classified as outliers after topic modeling (these were excluded from further analyses, see also Table S1).

### Frequency of life event topics

The topic modeling resulted in 12 distinct life event topics (see Figure 2). These were grouped into five thematically related higher‐order topics: education and career development (*n* = 2,873; 51.1%), social relationships (*n* = 1,101; 19.6%), leisure activities and successes (*n* = 697; 12.4%), mental health and well‐being (*n* = 629; 11.2%), and other life transitions and independence (*n* = 320; 5.7%; see Figure S2). The four most mentioned topics within these higher‐order topics were related to school, education, and apprenticeship (*n* = 2,504, 44.6%), romantic relationships and friendships (*n* = 687, 12.2%), mental health, development and change (*n* = 443, 7.9%), and traveling, vacation, and staying abroad (*n* = 415, 7.4%) (see Figure 3 and Table S1). All topics and their content are described in detail in Table S1, including example narratives of differing valence across ages 15, 17, 20, and 24 (see Table S1).

**Figure 3:**
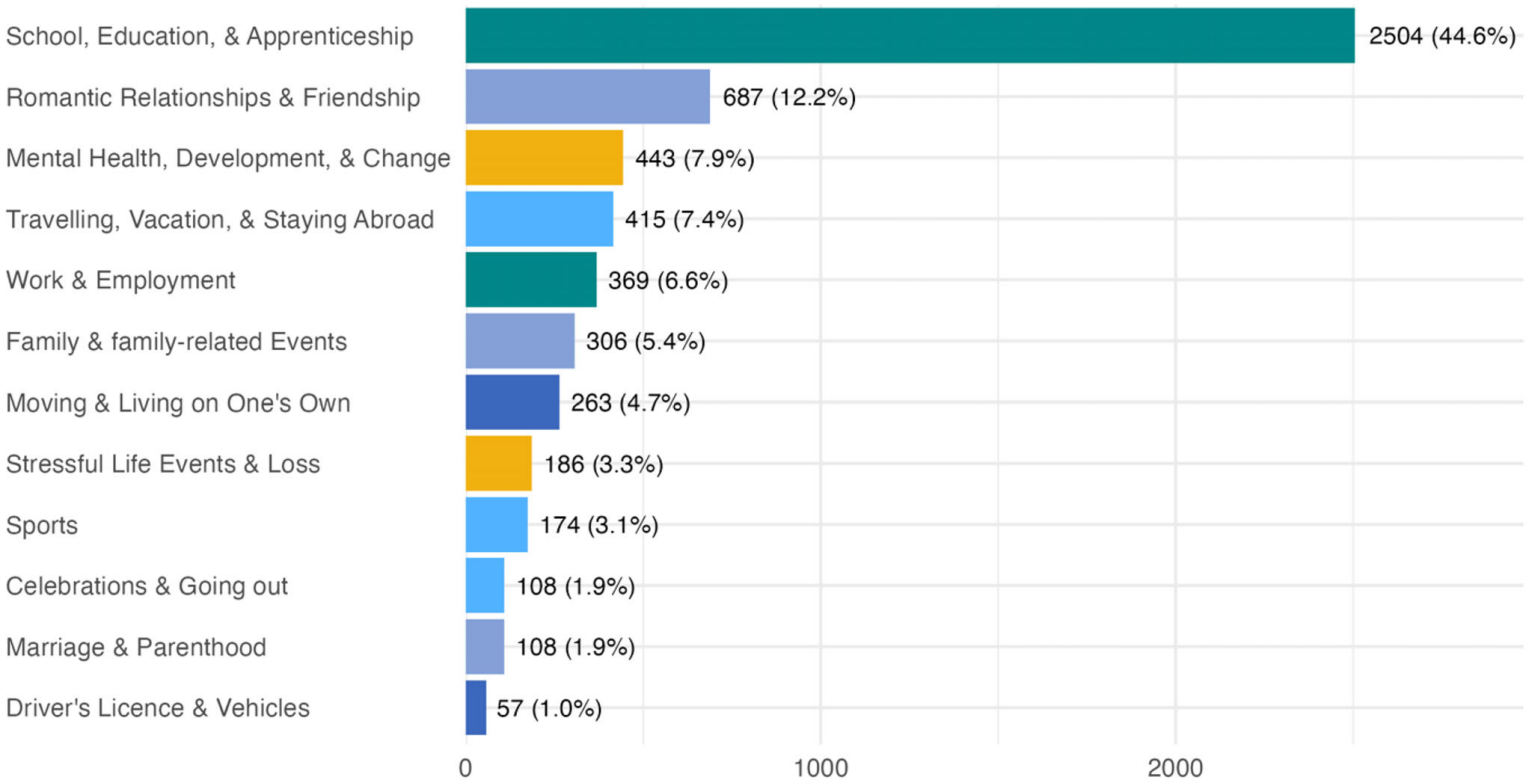
Frequency plot of life event topics. The figure displays the frequency of the 12 topics across all participants and measurement timepoints. The 12 topics are plotted on the *y*‐axis, and topic frequency across participants (i.e., how many participants mentioned a given topic) is plotted on the *x*‐axis. The color scheme indicates a topic's classification into one of five higher‐order categories: (1) education and career development (turquoise), (2) mental health and well‐being (yellow), (3) social relationships (mid/gray blue), (4) leisure activities and successes (sky blue), and (5) other life transitions and independence (dark blue)

### Emotional valence of life events

Most life events were classified as positive (*n* = 4,668, 83.1%), with the rest classified as either ambivalent/unclear (*n* = 597, 10.6%) or negative (*n* = 6.3%; see Figure S3). The valence of life events was unequally distributed across life event topics (*n* = 5,620, *df* = 22, Cramer's *V* = 0.57, Fisher's *p* < .001). While most life event topics were largely positive, the life event narratives within the topic stressful life events and loss were almost entirely classified as negative, and most narratives within the moving and living on one's own topic were classified as ambivalent/unclear (see Figure S4). Similarly, valence across higher‐order topics was unequally distributed (*n* = 5,620, χ^2^ = 1885.02, df = 8, Cramer's *V* = 0.41, *p* < .001; see Figure S5).

### Developmental shifts in life event topics

Stratifying life event topics across ages revealed distinct developmental shifts from midadolescence to young adulthood (see Figure 4). For example, the topic of school, education, and apprenticeship increased in frequency from age 15 to age 20, with work and employment continuously increasing in frequency from adolescence into young adulthood. In contrast, sports and celebrations and going out were reported less frequently with increasing age. Other topics, such as driver's license and vehicles, moving and living on one's own, and marriage and parenthood, were more frequently reported at ages 20 and 24. Logistic regression models, with sociodemographic factors and including age‐specific effects per topic, are presented in Tables S2 and S3. Adding age as a predictor increased the overall proportion of explained variance, with the models now explaining the range in Tjur's *R* ^2^ from 0.2% to 7.3%. Similarly, shifts in higher‐order life event topics are apparent from adolescence to young adulthood and displayed in Figure S6.

**Figure 4:**
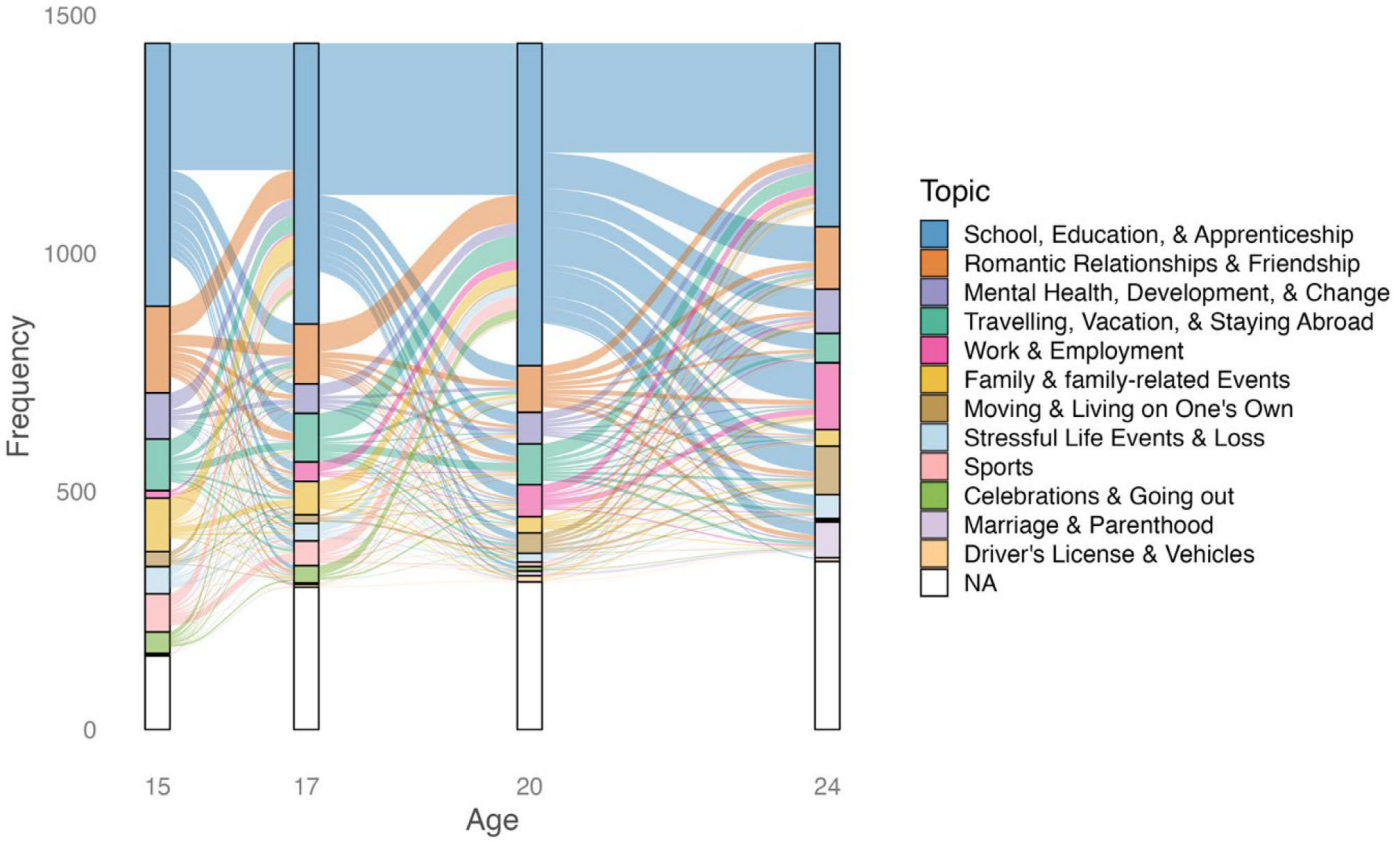
Alluvial plots of life event topics and shifts in topics across measurement timepoints from adolescence (ages 15, 17) into young adulthood (ages 20, 24). Measurement timepoints/ages are plotted on the *x*‐axis, frequency of topics is plotted on the *y*‐axis. Topics are sorted along their overall frequency from most frequent life event topic (School, Education, and Apprenticeship) toward least frequent life event topic (Driver's License and Vehicles). We included participants that contributed text data on at least one measurement timepoint of the cohort study. Thus, NAs are individuals who did not contribute text data at that specific timepoint. Larger NA proportions at later waves are due to cohort attrition

### Sociodemographic predictors of life event topics

To test the association between sociodemographic characteristics and life event topics, we fitted 12 logistic regression models (estimated using Maximum Likelihood) with cluster‐robust standard errors to predict each topic as a function of sex, SES, and migration background. The results of all models are displayed in Figure 5 and Table S2. In these models, women were significantly more likely to report life events related to traveling, vacation, and staying abroad, family and family‐related events, moving and living on one's own, stressful life events and loss, and marriage compared to men. In contrast, men had increased odds of reporting life events in the categories of sports and driver's license and vehicles compared to women. Higher SES was associated with higher odds of reporting life events within the topic traveling, vacation, and staying abroad and with lower odds of reporting on the topics of work and employment, family and family‐related events, celebrations and going out, and driver's license and vehicles. A family migration background was associated with higher odds of reporting the topics family and family‐related events and marriage and with lower odds of reporting on the topic moving and living on one's own (see Figure 5 and Table S2 (a–l)). No significant associations emerged between these three sociodemographic predictors and the three most common life event topics (school, education, and apprenticeship; romantic relationships and friendship; and mental health, development, and change) or for the topic celebrations and going out and parenthood. While these specific sociodemographic predictors reached significance, the overall proportion of explained variance was very small across all models (Tjur's *R* ^2^ values between 0.0% and 1.5%).

**Figure 5:**
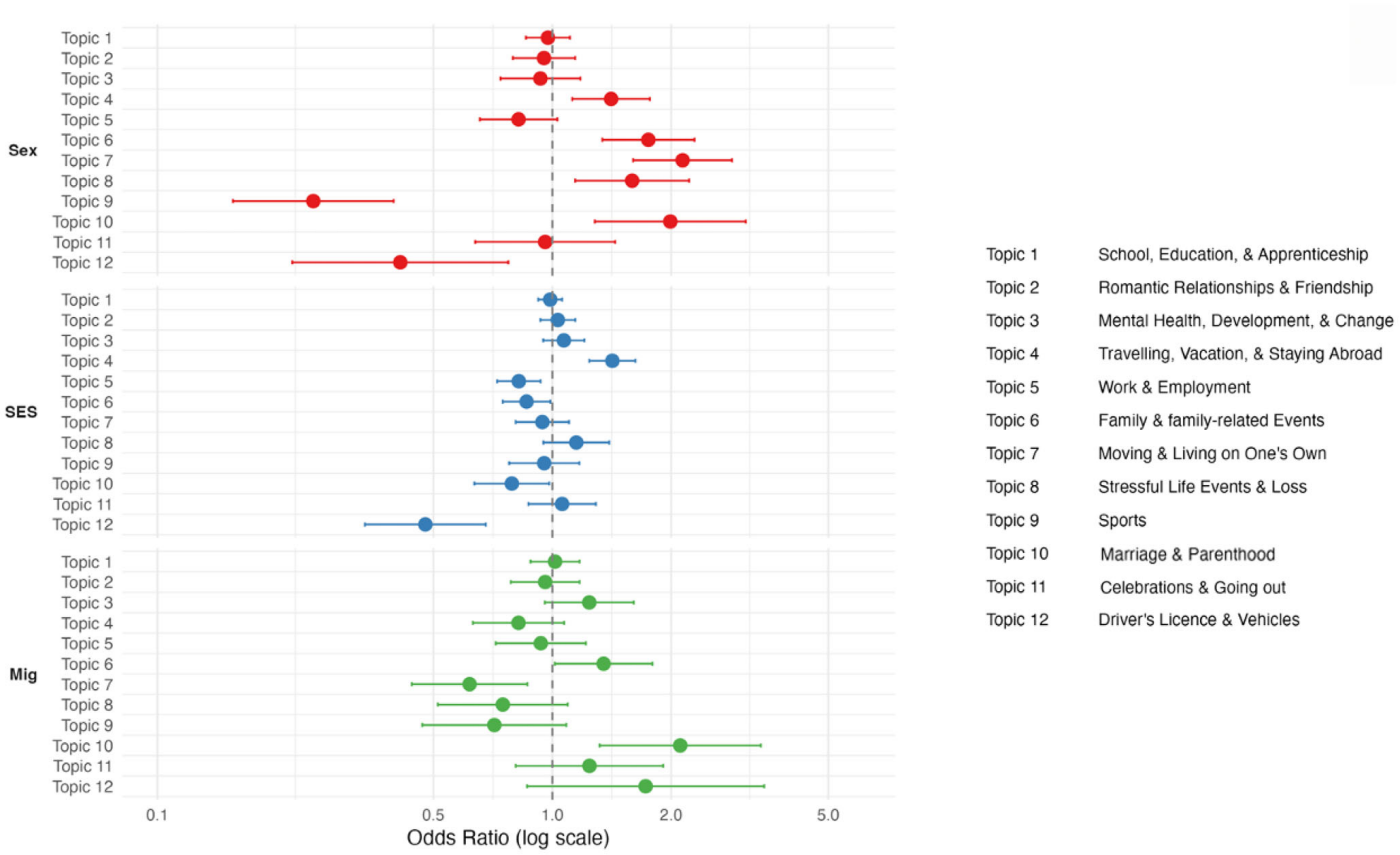
Sociodemographic predictors of life event topics. Estimates and corresponding 95%‐confidence intervals result from 12 multivariable binomial logistic regressions modeling specific life event topics as a function of sex (0 = male, 1 = female), SES (dimensional and standardized), and migration background (Mig; 0 = one parent Swiss born, 1 = both parents non‐Swiss born). Effects of the sociodemographic predictors are thus controlled for one another. Cluster‐robust standard errors were used to control for clustering of data within individuals

### Associations between internalizing symptoms with life event topics and valence

Logistic regression models examined whether current anxiety/depression symptoms were associated with life event topics, adjusting for sex, SES, migration background, and age. Higher anxiety/depression symptoms were associated with greater odds of reporting romantic relationships & friendship (OR = 1.20, 95% CI [1.09, 1.31], *p* < .001), mental health, development, & change (OR = 1.20, 95% CI [1.07, 1.35], *p* = .002), family & family‐related events (OR = 1.17, 95% CI [1.03, 1.34], *p* = .020), and stressful life events & loss (OR = 1.34, 95% CI [1.18, 1.62], *p* < .001) as the most important life event. In contrast, higher anxiety/depression symptoms were linked to lower odds of reporting school, education, & apprenticeship (OR = 0.90, 95% CI [0.84, 0.96], *p* = .002), traveling, vacation & staying abroad (OR = 0.83, 95% CI [0.74, 0.94], *p* = .003), work & employment (OR = 0.81, 95% CI [0.71, 0.92], *p* = .002), and sports (OR = 0.67, 95% CI [0.52, 0.86], *p* = .002) as the most important life event. A logistic regression with negative life event valence as outcome, adjusting for sociodemographic factors, showed that higher anxiety/depression was associated with increased odds of reporting a negative life event (OR = 1.52, 95% CI [1.34, 1.72], *p* < .001). Conversely, in a model with positive life event valence as outcome, higher anxiety/depression symptoms were associated with decreased odds of reporting a positive life event (OR = 0.80, 95% CI [0.73, 0.88], *p* < .001; see Figure S7; Tables S4 and S5).

## Discussion

This study is the first to examine openly assessed life events in adolescence and young adulthood using a state‐of‐the‐art NLP‐based topic modeling approach and leveraging data from a large urban longitudinal study. Adolescents and young adults reported a broad range of mostly positive, ‘normative’, and commonly occurring life events across multiple life domains (education and career development, social relationships, leisure activities and successes, mental health and well‐being, and other life transitions and independence). A distinct shift in topic frequency was displayed across development from adolescence to young adulthood, with sociodemographic characteristics including sex, SES, and migration background increasing the likelihood of reporting certain life event topics. Higher internalizing symptoms were associated with identifying more interpersonal and negatively valenced, and fewer positive or achievement‐related life events as most important.

Overall, the reported life event topics align well with developmental theory on transitions in adolescence and emerging adulthood, for example, events related to educational and occupational development, social and intimate relationships, or changes in habitation and mobility (Arnett, 2023; Arnett & Mitra, 2018; Graves & Larkin, 2006; Seiffge‐Krenke & Gelhaar, 2008). While school, education, and apprenticeship‐related events were most frequently reported in our study (44.6%), such topics are not commonly included in conventional life event checklists (except for entering college). Additionally, these checklists often fail to adequately cover educational paths that are uncommon in many English‐speaking countries, such as apprenticeships.^1^ Moreover, romantic relationship and friendship development are not always routinely represented in life event research. Life events checklists, for instance, typically include items on relationship loss or dissolution (e.g., breakups, separations), but rarely capture the emergence and unfolding of romantic relationships or close friendships as developmental tasks in their own right. In our study, however, such relational experiences were described as one of the most important topics (12.2%). Alongside these classic developmental transitions and milestones reported as important life events, topics also included diverse themes related to personality and identity development, change, self‐discovery, and episodes of poor mental health, highlighting important developmental processes of psychoemotional maturation in adolescence and young adulthood (Sawyer et al., 2018). Moreover, young people reported distinct major stressful and aversive experiences (e.g., loss, illness, and accidents), which are commonly investigated stressful life events (Bonanno, 2004; Cohen et al., 2019; Monroe & Slavich, 2019). This study exemplifies how artificial intelligence can help give people a voice and generate new insights from their experiences. Our results demonstrate that text‐based descriptions of life events can add unique information, context, or detail that complement and extend the scope of conventional self‐report life event checklists. Young people's reports of achieving positive developmental milestones highlight the importance of socioenvironmental support in attaining them.

It is particularly noteworthy that most major events reported by adolescents in our study were positive in valence, 'normative' in the sense that most individuals likely experience a variant of them as part of growing up, and seemingly 'mundane' in nature. This contrasts with much life event research and with many widely used checklists, which focus primarily on stressful or threatening events and therefore implicitly embed negative valence in item content. In contrast, more neutral life event inventories often do not assess perceived valence (Dohrenwend, 2006; Monroe & Slavich, 2019). The predominance of positively valanced yet normatively occurring events in our data underscores the need for more systematic attention to such experiences and their potential promotive and protective roles (Nunez‐Regueiro & Nunez‐Regueiro, 2021). While this pattern is especially evident for positive life events, many of the events reported within the ‘stressful life events and loss’ topic were not random or extraordinary traumas but rather developmentally normative experiences, such as the loss of a grandparent between ages 15 and 24. Similarly, events that may appear to be of low‐magnitude or ‘mundane’ from an adult perspective (e.g., the loss of a pet) can be experienced as highly distressing and consequential by adolescents (Copeland, Keeler, Angold, & Costello, 2010).

Beyond this broader pattern of ‘normative’ and seemingly ‘mundane’ events, our findings also speak to the potential promotive and protective role of positive experiences (Han et al., 2023; Morris et al., 2021; Narayan et al., 2023; Yarrington et al., 2023). In particular, the higher‐order topic leisure activities and successes (including travel, sports, and celebrations) stood out as distinct from all other topics. Such events can be highly meaningful, positive experiences for youth but are commonly excluded from life event checklists, nor are they often the focus of the youth mental health literature. This finding aligns well with youths' highlighting of positive changes during the COVID‐19 lockdown, including more time for leisure, recreation, and social interaction (Shanahan et al., 2023; Silk et al., 2022; Soneson et al., 2023). Positive experiences provide young people with important opportunities for joy, achievement, and personal growth, which are key for identity formation and social integration (Eccles, Barber, Stone, & Hunt, 2003; Hilbers, 2006; Zimmermann & Neyer, 2013). More specifically, travel exposes youth to new cultures and perspectives, promotes their independence, problem‐solving, and self‐discovery (Gallagher & Blythe, 2020); sports promote physical health and mental well‐being and teach teamwork, discipline, and goal‐setting (Brown, Patel, & Darmawan, 2017); and celebrations commonly mark important milestones and transitions, reinforcing cultural and family bonds and providing positive memories to later draw upon (Hilbers, 2006; Malaquias, Crespo, & Francisco, 2015). Identifying, creating, and leveraging positive experiences may be an important strategy within and beyond therapeutic contexts and may enhance the well‐being of young people (Sousa et al., 2025). While these implications align with positive psychological and developmental theory and the correlational patterns observed in our study, future research is needed to further investigate these assumptions.

Our study revealed significant sociodemographic differences and developmental shifts in the life events reported by adolescents and young adults. Our findings reveal distinct developmental shifts in life event topics from adolescence to young adulthood, aligning with established theories of psychosocial development (Arnett, 2023; Arnett & Mitra, 2018; Graves & Larkin, 2006; Seiffge‐Krenke & Gelhaar, 2008). The increasing frequency of education, work, and independence‐related events from ages 15 to 24 reflects the progression toward adult roles and responsibilities (Arnett, 2023; Arnett & Mitra, 2018). Conversely, the decrease in leisure‐related events suggests a reprioritization of time and energy as individuals navigate these transitions. Furthermore, we found that sex, SES, and migration background were significantly associated with the types of events experienced. However, the three most common topics (accounting for ~65% of all text entries) were unrelated to all predictors. Thus, while we found certain disparities for specific openly reported self‐selected life events, the effects were small to nonexistent, highlighting that most self‐selected, self‐reported life events generalize across social groups. Together, our results underscore the importance of considering salient age‐specific experiences and transitions when studying youth development and designing interventions to support successful transitions into adulthood.

Our work further suggests that internalizing symptoms are closely intertwined with which life events young people select as most important and with how positively or negatively these experiences are appraised and recalled. Higher internalizing symptoms may increase the exposure to particular events via stress‐generation processes (Jenness, Peverill, King, Hankin, & McLaughlin, 2019; Rnic et al., 2023), whereby depression‐related vulnerabilities may contribute to more frequent exposure to or worry about interpersonal and other dependent stressors (e.g., breakups of relationships) (Haag et al., 2026) or hinder certain positive experiences (e.g., to travel or to go out). At the same time, stressful life events are well‐established predictors of subsequent internalizing problems (e.g., illness or death of spouses), implying that some of the reported events may themselves be risk factors for later symptoms (Lecarie et al., 2022; March‐Llanes, Marques‐Feixa, Mezquita, Fananas, & Moya‐Higueras, 2017). Finally, mood‐congruent memory and appraisal biases are likely to influence which events are selected and retrieved, with youth experiencing internalizing symptoms more inclined to foreground negative or threatening aspects of their lives and to less likely report positive experiences (Barry, Hallford, & Takano, 2021; Faul & LaBar, 2023). Together, our findings highlight theory‐consistent patterns of bidirectional associations between symptoms, exposure to specific types of life events, and the valence of these events. Future research should investigate the dynamic interplay of openly queried life events with internalizing symptoms, to clarify their temporal dynamics, and to test if fostering beneficial experiences may reduce internalizing problems.

Our work has several limitations. First, the cognitive processes that guide the selection of significant life events are unclear. The timing of the data collection periods (between early spring and summer) might have resulted in certain life events being recalled more frequently, such as events related to school or educational transitions. Moreover, participants' current mood may impact the recall of salient life events, as shown in previous work regarding depressive or anxious individuals (McFadden & Siedlecki, 2020; Ramsgaard, Bohn, & Thastum, 2019). Second, participants were asked to describe only their single most important life event at each assessment. This approach may have limited the breadth and representativeness of captured experiences, potentially omitting events that were subjectively meaningful but less salient at the time of reporting. Nevertheless, the emergence of coherent and interpretable topics despite these constraints underscores the feasibility and added value of applying NLP methods in settings with even limited text data. Third, the analytical pipelines for such topic modeling analyses are under active development, without agreed‐upon standards or workflows. Certain analytical decisions may have influenced the results (e.g., size of clusters and probability threshold for cluster assignment during clustering). Furthermore, smaller sample sizes may limit the detection of rare or highly specific topics, as text segments referring to such topics may occur only infrequently and therefore not form stable clusters; examples of these low‐frequency topics are provided in Table S1. Moreover, we used coded emotional valence data from age 15 as training data to fine‐tune a transformer model‐based classifier. These data had been previously coded shortly after the original paper–pencil data was entered into the database according to face validity. After fine‐tuning the classifier, we classified all text entries using this model. To increase the validity of our approach, we conducted a manual review of all training data, classified valence ratings across the entire database, and corrected misclassified life events. Fourth, while age 15 was largely representative of the initial target sample (Eisner et al., 2018; Ribeaud et al., 2022), selective attrition at ages 17, 20, and 24 (e.g., lower participation among men, participants from lower SES backgrounds, and those with a migration background) may limit representativeness. Last, our data were collected between 2013 and 2022 in the urban region of Zurich, Switzerland. While most of the life events reported represent common experiences during adolescence and young adulthood, others are time‐ and place‐dependent—for example, Justin Bieber concerts, the COVID‐19 pandemic, or the Russian war on Ukraine—and thus reflect time‐, place‐, or age‐related experiences which do not directly generalize to other times, contexts, and populations.

Nevertheless, our work has important strengths. We used a novel state‐of‐the‐art topic modeling approach combining LLMs with conventional techniques to study youths' self‐reported, openly queried, most important life events. We drew on open‐text data from a large multiethnic urban cohort across four measurement timepoints from adolescence into young adulthood (ages 15–24), covering important developmental phases. Together, the novel methods, strong longitudinal research design, combination of text data with sociodemographic data, and timely research approach and question make this an important contribution to the youth mental health field and a methodological blueprint for other work using computational‐qualitative methods.

Future application of such methods in risk and resilience and youth mental health research could include studying further topics fostering recent uncertainty and loneliness among young people, such as for instance their hopes and worries looking into the future (Haag et al., 2026; Rohrer et al., 2017), or reasons for social and societal disengagement. In particular, analyzing subjective text‐based accounts at scale might help to further investigate intersecting risk factors in specific vulnerable groups and to elucidate salient promotive and protective factors among these youth. Future studies may also benefit from collecting multiple self‐identified life events, ranking them by importance, and incorporating dimensional ratings of their specific emotional valence. Such designs would allow for a broader assessment of the relative importance and affective tone of youths' experiences and could investigate whether early adversity predicts a higher burden of negative and fewer positive self‐reported life events across development. Moreover, future studies could explicitly combine investigator‐defined checklists with open‐text reports by mapping checklist items onto inductively derived topics and classifying free‐text responses according to existing scales to explicitly display divergence between standardized life event measures and self‐defined major events that are salient for youth. Beyond single studies, LLMs could also support metascientific work by systematically analyzing the item content of widely used life event scales, extracting common themes, and classifying their valence. Such analyses would clarify which domains and valence patterns are over‐ or underrepresented in current instruments and how these compare to self‐defined events reported by young people (Wulff & Mata, 2025). Ultimately, such insights from text‐based analyses might help to develop more targeted and effective interventions, tailoring support mechanisms based on the specific needs and perspectives expressed by young people themselves.

## Conclusion

Most young people reported positive, 'normative', and commonly occurring life events as their most important life events in the past two to four years. These topics covered diverse life domains (such as education and career development, social relationships, leisure activities and successes, mental health and well‐being, and other life transitions and independence). Our findings thus contribute to a deeper understanding of diverse topics related to self‐reported life events at different ages. Moreover, our research provides a flexible blueprint for using open‐text data to complement common quantitative research methods in an inductive, bottom‐up discovery‐science approach, illustrating how longitudinal population‐based research can draw on large‐scale text data through NLP techniques. Importantly, our findings highlight that, in addition to helping young people cope with major life stressors, the promotion of beneficial experiences, leisure activities, and successes may foster well‐being and productive youth development.

## Ethical considerations

The data used in this study are covered by two ethics approvals for the z‐proso study: Phase 5 (2017–2020; approval number: 2018.2.12) and Phase 6 (2021–2024; approval number: 21.12.13), both from the Ethics Committee of the Faculty of Arts and Social Sciences, University of Zurich. In accordance with Swiss Law, participants provided informed consent at all measurement timepoints (ages 15, 17, 20, and 24). At age 15, parental passive consent was obtained (parents could refuse participation of their children).

**Key points**

**What's known?**

- Major life events are among the most studied risk factors for psychopathology. However, population‐based studies on risk and resilience factors for youth mental health rarely include young people's subjective lived experience.

**What's new?**

- In this study, the large majority of young people's open text‐based accounts of their *most important life event* were positive, topics covered diverse life domains, and shifted as youth transitioned from adolescence to young adulthood.
- Higher anxiety and depression symptoms were associated with identifying more interpersonal and stressful life events as most important and fewer positive or achievement‐related ones.

**What's relevant?**

- Open‐text data and Natural Language Processing (NLP) techniques offer valuable tools for population‐based, longitudinal research on youth mental health, augmenting common quantitative research methods in an inductive and exploratory approach.

## Supporting information


**Appendix S1.** Complementary information of text preprocessing and the BERTopic analysis.
**Appendix S2.** Training an LLM‐based classifier for emotional valence.
**Figure S1.** Emotional valence classifier.
**Figure S2.** Higher‐order topics frequencies.
**Figure S3.** Life events valence.
**Figure S4.** Life event valence by topic.
**Figure S5.** Life event valence by higher‐order topics.
**Figure S6.** Alluvial plot of higher‐order topics transitions across ages.
**Figure S7.** Associations between symptoms of anxiety/depression with life event topics.
**Table S1.** Topic modeling results: topic description and text examples.
**Table S2.** Logistic Regression Model Outputs (corresponding to Figure 4).
**Table S3.** Logistic regression model outputs including sociodemographic factors and age.
**Table S4.** Logistic regression model outputs including sociodemographic factors, age and internalizing symptoms.
**Table S5.** Logistic regression model outputs of topic valence by anxiety/depression, SES, sex, migration background, and age.

**Appendix S3.** ALPACA BERTopic.

**Appendix S4.** ALPACA emotional valence classifier.

## Data Availability

The data that support the findings of this study are available upon reasonable request to the z‐proso Principal Investigators (D.R., L.S., and M.E.). Text data are not publicly available due to privacy restrictions. Other z‐proso data are successively becoming available through the SWISSUbase platform and can be requested by researchers (project link: https://www.swissubase.ch/de/catalogue/studies/9707/20382/overview). All users are required to sign a user agreement with the University of Zurich or with SWISSUbase to access the data.

## Endnote

1 The Swiss apprenticeship system, known as the dual‐track system, combines practical on‐the‐job training in companies with theoretical education in vocational schools, typically lasting three to four years. This system, which educates about 70% of Swiss upper secondary students, offers apprenticeships in approximately 250 professions and provides a flexible pathway for further education, including access to universities of applied sciences (Nancy Hoffmann for Jobs for the Future, 2018).
