# Supplementary material for: Personally meaningful life events from adolescence to young adulthood: a longitudinal natural language processing analysis
Source: J Child Psychol Psychiatry. 2026 Jun 22;67(12):1937–52. doi: 10.1111/jcpp.70169 (PMC13745790; doi:10.1111/jcpp.70169)
Supplement: Supplementary file 1 [file JCPP-67-1937-s001.pdf]

In [2]:
```
####################################
#                                  #
#       LLM-BASED CLASSIFIER       #
#      FOR EMOTIONAL VALENCE       #
#                                  #
####################################


### This code is based on the following Hugging Face Tutorial:
### https://huggingface.co/docs/transformers/training


# Import libraries
import pandas as pd
from datasets import Dataset

# Load files
df = pd.read_excel("/data/ALPACA/ALPACA_1_finalized_file_6.4.25.xlsx")
# Subset rows where age is 15
df = df[df['age'] == 15]


# Renaming variables
df = df.rename(columns={
    'valence': 'label',
    'Document': 'text'
})
# Check label variable
print(df['label'].unique())
```

```
['Positive' 'ambivalent/unclear' 'Negative']
```

In [3]:
```
# Recode label variable to numeric
# Define the mapping: start at 0
mapping = {
    'Positive': 0,
    'ambivalent/unclear': 1,
    'Negative': 2
}
```

```
# Apply the mapping
df['label'] = df['label'].map(mapping)
# Check if this worked
print(df['label'].unique())
```

```
[0 1 2]
```

In [5]:

```
# Splitting the dataframe into training and validation data
# while stratifying for valence categories

from sklearn.model_selection import train_test_split

train_df, val_df = train_test_split(
    df,
    test_size=0.2,
    stratify=df['label'],  # ensures even category distribution
    random_state=42        # reproducibility
)

# Convert to Hugging Face dataset format
train = Dataset.from_pandas(train_df)
test  = Dataset.from_pandas(val_df)


# Tokenize data
from transformers import AutoTokenizer
tokenizer = AutoTokenizer.from_pretrained('/data/GermanBERTuncased')


def tokenize_function(examples):
    return tokenizer(examples["text"], padding="max_length", truncation=True)

train = train.map(tokenize_function, batched=True)
test  = test.map(tokenize_function, batched=True)
```

```
Map: 100%|██████████| 1155/1155 [00:00<00:00, 4025.05 examples/s]
Map: 100%|██████████| 289/289 [00:00<00:00, 3469.75 examples/s]
```

In [6]:

```
# Load German BERT model (stored locally and loaded from disk)
# num_labels = needs to be set to the number of unique labels
```

```
from transformers import AutoModelForSequenceClassification

path = '/data/GermanBERTuncased'
model = AutoModelForSequenceClassification.from_pretrained(path, num_labels=3)
```

```
Some weights of BertForSequenceClassification were not initialized from the model checkpoint at /data/      GermanB
ERTuncased and are newly initialized: ['classifier.bias', 'classifier.weight']
You should probably TRAIN this model on a down-stream task to be able to use it for predictions and inference.
```

In [7]:

```
#  Training hyperparameters
from transformers import TrainingArguments, Trainer
import numpy as np
from sklearn.metrics import precision_recall_fscore_support, accuracy_score


def compute_metrics(eval_pred):
    logits, labels = eval_pred
    preds = np.argmax(logits, axis=-1)
    acc = accuracy_score(labels, preds)
    precision, recall, f1, _ = precision_recall_fscore_support(
        labels, preds, average='macro'
    )
    return {
        "eval_accuracy": acc,
        "eval_f1_macro": f1,
        "eval_precision_macro": precision,
        "eval_recall_macro": recall,
    }


# Monitoring evaluation metrics during fine-tuning
training_args = TrainingArguments(output_dir="/data/ALPACA/",
                                  eval_strategy="epoch",
                                  logging_dir='/data/ALPACA/logs',
                                  logging_steps=10,
                                  num_train_epochs=10,
                                  save_strategy="epoch",
                                  load_best_model_at_end=True,       # Load the best model based on evaluation metr
                                  metric_for_best_model="f1_macro",  # Metric to determine the best model
                                  greater_is_better=True  # For F1 score, higher is better
```

```
                                  )


# Prepare trainer
trainer = Trainer(model=model,
                  args=training_args,
                  train_dataset=train,
                  eval_dataset=test,
                  compute_metrics=compute_metrics)
```

In [8]:
```
# Run classifier training

trainer.train()
```

[1450/1450 06:34, Epoch 10/10]

Epoch	Training Loss	Validation Loss	Accuracy	F1 Macro	Precision Macro	Recall Macro
1	0.340900	0.305488	0.910035	0.759944	0.867252	0.705422
2	0.535600	0.259209	0.920415	0.849542	0.863757	0.836900
3	0.193100	0.488130	0.910035	0.815777	0.821680	0.820233
4	0.262700	0.658509	0.896194	0.754172	0.845296	0.713355
5	0.120800	0.578980	0.913495	0.822200	0.854345	0.796689
6	0.000500	0.542336	0.916955	0.815596	0.867968	0.776055
7	0.000300	0.586324	0.913495	0.811577	0.860473	0.774600
8	0.000400	0.526450	0.913495	0.817603	0.861134	0.782933
9	0.028100	0.545219	0.920415	0.829739	0.888317	0.785844
10	0.000200	0.546930	0.916955	0.825755	0.880667	0.784389

Out[8]:
```
TrainOutput(global_step=1450, training_loss=0.10840801017005638, metrics={'train_runtime': 395.6353, 'train_sample
s_per_second': 29.194, 'train_steps_per_second': 3.665, 'total_flos': 3038959974758400.0, 'train_loss': 0.10840801
017005638, 'epoch': 10.0})
```

In [9]:
```
# After training, the best model is automatically loaded and can be saved

model.save_pretrained("/data/ALPACA/best_model")
```

In [1]:
```
########################
#                      #
# Applying the trained #
#     classifier to    #
#       new data       #
#                      #
########################

import torch
from transformers import AutoTokenizer, AutoModelForSequenceClassification
import pandas as pd

# Load tokenizer and model
tokenizer = AutoTokenizer.from_pretrained('/data/ALPACA/best_model')
model = AutoModelForSequenceClassification.from_pretrained('/data/ALPACA/best_model')

# Set the model to evaluation mode
model.eval()
```

```
/data/chhaag/conda/envs/myenv3/lib/python3.11/site-packages/tqdm/auto.py:21: TqdmWarning: IProgress not found. Pleas
e update jupyter and ipywidgets. See https://ipywidgets.readthedocs.io/en/stable/user_install.html
  from .autonotebook import tqdm as notebook_tqdm
```

Out[1]:
```
BertForSequenceClassification(
  (bert): BertModel(
    (embeddings): BertEmbeddings(
      (word_embeddings): Embedding(31102, 768, padding_idx=0)
      (position_embeddings): Embedding(512, 768)
      (token_type_embeddings): Embedding(2, 768)
      (LayerNorm): LayerNorm((768,), eps=1e-12, elementwise_affine=True)
      (dropout): Dropout(p=0.1, inplace=False)
    )
    (encoder): BertEncoder(
      (layer): ModuleList(
        (0-11): 12 x BertLayer(
          (attention): BertAttention(
            (self): BertSdpaSelfAttention(
              (query): Linear(in_features=768, out_features=768, bias=True)
              (key): Linear(in_features=768, out_features=768, bias=True)
              (value): Linear(in_features=768, out_features=768, bias=True)
              (dropout): Dropout(p=0.1, inplace=False)
            )
            (output): BertSelfOutput(
              (dense): Linear(in_features=768, out_features=768, bias=True)
              (LayerNorm): LayerNorm((768,), eps=1e-12, elementwise_affine=True)
              (dropout): Dropout(p=0.1, inplace=False)
            )
          )
          (intermediate): BertIntermediate(
            (dense): Linear(in_features=768, out_features=3072, bias=True)
            (intermediate_act_fn): GELUActivation()
          )
          (output): BertOutput(
            (dense): Linear(in_features=3072, out_features=768, bias=True)
            (LayerNorm): LayerNorm((768,), eps=1e-12, elementwise_affine=True)
            (dropout): Dropout(p=0.1, inplace=False)
          )
        )
      )
    )
    (pooler): BertPooler(
      (dense): Linear(in_features=768, out_features=768, bias=True)
      (activation): Tanh()
```

```
      )
    )
    (dropout): Dropout(p=0.1, inplace=False)
    (classifier): Linear(in_features=768, out_features=3, bias=True)
  )
```

In [6]:
```
# Define function to classify emotional valence

def classify_valence(sentence):
    try:
        # Tokenize the sentence
        inputs = tokenizer(sentence, return_tensors="pt", padding=True, truncation=True, max_length=512)
        # Make prediction
        with torch.no_grad():
            outputs = model(**inputs)
            logits = outputs.logits
        # Get the predicted class
        predicted_class = torch.argmax(logits, dim=-1).item()
        sentiment_labels = {0: 'Positive',
                            1: 'ambivalent/unclear',
                            2: 'Negative'}
        return sentiment_labels[predicted_class]
    except Exception as e:
        print(f"Error classifying text: {sentence}")
        print(e)
        return None
```

In [7]:
```
# Load data

df = pd.read_excel("/data/ALPACA/ALPACA_1_finalized_file_6.4.25.xlsx")


# Apply classifier to text data variable only to rows where age != 15
# and store predicted value per row in the valence variable
df.loc[df['age'] != 15, 'valence_pred'] = df.loc[df['age'] != 15, 'Document'].apply(lambda x: classify_valence(x))


# Save to disk
```

```
df.to_excel('/data/ALPACA/ALPACA_predicted_valence.xlsx')
```

In [10]:

```
# Load data after manual correction:
# Manually corrected data are saved in column "valence"
df_reviewed = pd.read_excel('/data/ALPACA/ALPACA_predicted_valence_reviewed.xlsx')

# How many values were corrected manually?
# Filter the relevant rows
filtered_df = df_reviewed[df_reviewed['age'] != 15].copy()

# Compute agreement (True if values match, False if not)
filtered_df['agreement'] = filtered_df['valence'] == filtered_df['valence_pred']

# Agreement between predictions and manual corrections:
print(filtered_df['agreement'].value_counts())
```

```
agreement
True     3694
False     533
Name: count, dtype: int64
```

In [12]:

```
# Create a cross-tabulation of 'var' and 'valence'
disagreement_table = pd.crosstab(filtered_df['agreement'], filtered_df['valence'])

# Display the result
print(disagreement_table)
```

```
valence    Negative  Positive  ambivalent/unclear
agreement
False            82       345                 106
True            172      3220                 302
```

In [13]:

```
# Create a DataFrame that shows where changes happen
filtered_df['valence_change'] = filtered_df['valence_pred'] != filtered_df['valence']

# Find the most frequent changes (transitions)
change_counts = pd.crosstab(filtered_df['valence_pred'], filtered_df['valence'])

# Display the result
print(change_counts)
```

valence	Negative	Positive	ambivalent/unclear
valence_pred			
Negative	172	53	28
Positive	46	3220	78
ambivalent/unclear	36	292	302
