# Supplementary material for: Personally meaningful life events from adolescence to young adulthood: a longitudinal natural language processing analysis
Source: J Child Psychol Psychiatry. 2026 Jun 22;67(12):1937–52. doi: 10.1111/jcpp.70169 (PMC13745790; doi:10.1111/jcpp.70169)
Supplement: Supplementary file 2 [file JCPP-67-1937-s003.pdf]

In [12]:

```
####################################
#                                  #
#     Topic Modelling Analysis     #
#           using BERTopic         #
#                                  #
####################################


### The following code is based on the guidelines and examples provided on the BERTopic website:
### https://maartengr.github.io/BERTopic/algorithm/algorithm.html


# Load modules
import pandas as pd
import re
import bertopic
from bertopic import BERTopic
import sentence_transformers
from sentence_transformers import SentenceTransformer
import umap
from umap import UMAP
from bertopic.vectorizers import ClassTfidfTransformer
from bertopic.representation import MaximalMarginalRelevance
from hdbscan import HDBSCAN
import spacy
import numpy as np
import nltk
nltk.data.path.append('/data/nltk_data')
from nltk.corpus import stopwords
import sklearn
from sklearn.feature_extraction.text import CountVectorizer
import sys
import notebook
german_stop_words = stopwords.words('german')


# Print package versions
from importlib.metadata import version
print("hdbscan version:", version("hdbscan"))
```

```
print("re version:", re.__version__)
print("pandas version:", pd.__version__)
print("bertopic version:", bertopic.__version__)
print("sentence_transformers version:", sentence_transformers.__version__)
print("umap-learn version:", umap.__version__)
print("spacy version:", spacy.__version__)
print("numpy version:", np.__version__)
print("nltk version:", nltk.__version__)
print("scikit-learn version:", sklearn.__version__)
print("hdbscan version:", version("hdbscan"))

# Print the Python version
print("Python version:", sys.version)

# Print the Jupyter Notebook version
print("Jupyter Notebook version:", notebook.__version__)
```

```
hdbscan version: 0.8.41
re version: 2.2.1
pandas version: 3.0.0
bertopic version: 0.17.4
sentence_transformers version: 5.2.2
umap-learn version: 0.5.11
spacy version: 3.8.11
numpy version: 2.3.5
nltk version: 3.9.2
scikit-learn version: 1.8.0
hdbscan version: 0.8.41
Python version: 3.11.14 | packaged by conda-forge | (main, Jan 26 2026, 23:48:32) [GCC 14.3.0]
Jupyter Notebook version: 7.5.3
```

In [3]:

```
# Load data
df =  pd.read_excel('/data/ALPACA_BERTopic/alpaca_cleaned_LB.xlsx')

# Drop rows where 'text' is NaN
df = df.dropna(subset=['text'])

# Split the text at each dot and expand into new rows
df['text'] = df['text'].astype(str).str.split('.')
df = df.explode('text')
```

```
# Optionally, you can remove any leading or trailing spaces
df['text'] = df['text'].str.strip()

# Drop any empty rows if they exist (in case there were trailing dots)
df = df[df['text'] != '']

# Ensure 'text' is treated as a string, then drop rows where 'text' contains only numbers or only punctuation
df = df[df['text'].notna()]  # Remove NaN values
df = df[~df['text'].str.match(r'^\s*[\d\W]+\s*$')]


# Reducing noise by
df['text'] = df['text'].replace("Berufsmaturitätsschule", "BMS", regex=True)
df['text'] = df['text'].replace("Berufsmittelschule", "BMS", regex=True)
df['text'] = df['text'].replace("Fachmittelschule",  "FMS", regex=True)
df['text'] = df['text'].replace("Lehrabschlussprüfung",  "LAP", regex=True)
df['text'] = df['text'].replace("Handelsmittelschule",  "HMS", regex=True)
df['text'] = df['text'].replace("Gymnasium",  "Gymi", regex=True)
df['text'] = df['text'].replace("Kantonsschule",  "Gymi", regex=True)
df['text'] = df['text'].replace("Maturität",  "Matur", regex=True)
df['text'] = df['text'].replace("Matura",  "Matur", regex=True)
df['text'] = df['text'].replace("Kantiprüfung",  "Matur", regex=True)
df['text'] = df['text'].replace("Gymiprüfung",  "Matur", regex=True)


# Remove rows with NaN values
df = df.dropna(subset=['text'])

# Convert to list
events = df.text.to_list()

# Save id and age variables as lists as well for download
id = df.id.to_list()
age = df.age.to_list()
```

In [4]:

```
### BERTOPIC analysis procedure


# Extracting embeddings
model_embedding = SentenceTransformer("paraphrase-multilingual-MiniLM-L12-v2")
```

```
# Reducing dimensionality
umap_model = UMAP(n_neighbors=11, min_dist=0.0, random_state=1, init="random")


# Clustering the reduced embeddings
hdbscan_model = HDBSCAN(prediction_data=True)


# Tokenizing the text segments

# Expand stop words list and remove numbers
german_stop_words.extend(['jahr', 'monat', 'woche',
                          'januar', 'februar', 'märz', 'april', 'mai', 'juni', 'juli',
                          'august', 'september','oktober', 'november', 'dezember'])


# Lemmatizer
nlp = spacy.load("de_core_news_sm")
#
def lemma(text, allowed_postags=['NOUN']): # , 'ADJ','VERB','ADV'
    text = re.sub(r'\d+', '', text)
    text = re.sub(r'[,.!?+:-=`<>"/!()&%]', ' ', text)
    text = ' '.join([word.lemma_ for word in nlp(text) if word.pos_ in allowed_postags])
    text = text.lower()
    return text


#
vectorizer_model= CountVectorizer(stop_words=german_stop_words, preprocessor=lemma)


# Creating the topic representations
ctfidf_model = ClassTfidfTransformer(reduce_frequent_words=True)

# Fine-tuning topic representations with MNR
representation_model = MaximalMarginalRelevance(diversity=0.1)

# Training the BERTopic model:
topic_model = BERTopic(embedding_model=model_embedding,
```

```python
                        representation_model=representation_model,
                        vectorizer_model=vectorizer_model,
                        umap_model=umap_model,
                        ctfidf_model=ctfidf_model,
                        n_gram_range=(1,1),
                        min_topic_size=30,
                        calculate_probabilities=True)

#
topics, probabilities = topic_model.fit_transform(events)
```

```
Loading weights: 100%|██████████| 199/199 [00:00<00:00, 770.00it/s, Materializing param=pooler.dense.weight]
BertModel LOAD REPORT from: sentence-transformers/paraphrase-multilingual-MiniLM-L12-v2
Key                     | Status     |  |
------------------------+------------+--+-
embeddings.position_ids | UNEXPECTED |  |

Notes:
- UNEXPECTED    :can be ignored when loading from different task/architecture; not ok if you expect identical arch.
```

In [7]:

```python
### Displaying the top 10 keywords per topic and the outlier (-1) category

# Retrieve the topic info DataFrame
topic_info = topic_model.get_topic_info()

# Iterate through each topic, including topic -1
for index, row in topic_info.iterrows():
    topic = row.Topic
    freq  = row.Count  # Frequency of the topic
    # Retrieve the top keywords for the current topic (each element is (keyword, weight))
    keywords_with_weights = topic_model.get_topic(topic)

    # Show the top 3 keywords per topic
    top_3_keywords = keywords_with_weights[:3]

    # Extract just the keywords from the tuples
    keywords_only = [keyword for keyword, weight in top_3_keywords]

    print(f"Topic {topic} (Frequency: {freq}): {keywords_only}\n")
```

```
################################
### RESULTS IN 32 TOPICS

#'PLACEHOLDER_PII' = contains information e.g. about a place, an event or a name
```

Topic -1 (Frequency: 959): ['zuhause', 'depression', 'militärdienst']

Topic 0 (Frequency: 1674): ['sekundarschule', 'beruf', 'berufsleben']

Topic 1 (Frequency: 616): ['liebe', 'beziehung', 'freundschaft']

Topic 2 (Frequency: 382): ['persönlichkeit', 'gefühl', 'religion', 'ziel', 'lebensstil', 'perspektive', 'komfortzone']

Topic 3 (Frequency: 208): ['pandemie', 'suizidversuch', 'matur']

Topic 4 (Frequency: 170): ['ferien', 'reise', 'sommerferien']

Topic 5 (Frequency: 145): ['familie', 'scheidung', 'verlust']

Topic 6 (Frequency: 142): ['sport', 'PLACEHOLDER_PII', 'PLACEHOLDER_PII']

Topic 7 (Frequency: 122): ['gymi', 'fitness', 'fitnessabo']

Topic 8 (Frequency: 109): ['elternhaus', 'wohngemeinschaft', 'wohnsituation']

Topic 9 (Frequency: 87): ['jobanfang', 'lehranfang', '']

Topic 10 (Frequency: 78): ['geburtstag', 'schulwechsel', 'volljährigkeit']

Topic 11 (Frequency: 71): ['lehrbeginn', 'anfang', 'start']

Topic 12 (Frequency: 70): ['schwangerschaft', 'halbschwester', 'tatoo']

Topic 13 (Frequency: 59): ['bms', 'prüfung', 'fachhochschule']

Topic 14 (Frequency: 58): ['heirat', 'verlobung', 'liebeskummer']

Topic 15 (Frequency: 57): ['freundin', 'lehrstelle', '']

Topic 16 (Frequency: 56): ['cousine', 'onkel', 'cousin']

Topic 17 (Frequency: 56): ['lehrvertrag', 'unterzeichnung', 'detailhandelsfachfrau']

Topic 18 (Frequency: 52): ['familienmitglied', 'selbstmord', 'urgrossmütter']

```
Topic 19 (Frequency: 49): ['bank', 'lehrstellensuche', 'lehrstelle']

Topic 20 (Frequency: 48): ['autoprüfung', 'auto', 'fahrprüfung']

Topic 21 (Frequency: 39): ['lehrstell', 'suche', 'finde']

Topic 22 (Frequency: 38): ['jobwechsel', 'berufswechsel', 'berufsfeldwechsel']

Topic 23 (Frequency: 37): ['klassenlager', '', '']

Topic 24 (Frequency: 37): ['sprachaufenthalt', 'sprachaufenhtalt', 'sprachsemester']

Topic 25 (Frequency: 35): ['geburtstag', 'job', 'geburtstagsesse']

Topic 26 (Frequency: 34): ['lehrabschluss', 'konsum', 'brust']

Topic 27 (Frequency: 34): ['traumlehrstell', 'lehrstelle', 'schule']

Topic 28 (Frequency: 33): ['beginn', 'lehre', 'anfang']

Topic 29 (Frequency: 33): ['musik', 'band', 'gesang']

Topic 30 (Frequency: 32): ['zwischenjahr', 'assesmentjahr', 'fussoperation']

Topic 31 (Frequency: 31): ['auslandaufenthalt', 'auslandsaufenthalt', 'langzeitwanderung']
```

In [8]:

```
# Merging of overlapping topics

topics_to_merge = [[0, 7, 11, 13, 17, 19, 21, 23, 27, 28, 30],  # School, Education, Apprenticeship
                   [9, 22],                                     # Work, Employment
                   [3, 18],                                     # Stressful Life Events & Loss
                   [1, 15],                                     # Romantic Relationships & Friendships
                   [5, 16, 26],                                 # Family & Family-Related Events
                   [12, 14],                                    # Marriage & Parenthood
                   [4, 24, 31],                                 # Travelling, Vacation, Staying Abroad
                   [10, 25, 29]]                                # Celebrations & Going Out

                                                                ### Topics which are not being merged:
                                                                # Mental Health, Development & Change
                                                                # Sports
```

```
                                                                 # Moving & Living on One’s Own
                                                                 # Driver’s License & Vehicles



topic_model.merge_topics(events, topics_to_merge)



# Update topic and probabilities variables
import hdbscan
topics        = topic_model._map_predictions(topic_model.hdbscan_model.labels_)
probabilities = hdbscan.all_points_membership_vectors(topic_model.hdbscan_model)
probabilities = topic_model._map_probabilities(probabilities, original_topics=False)
```

In [9]:
```
### Displaying the top 10 keywords per topic and the outlier (-1) category

# Retrieve the topic info DataFrame
topic_info = topic_model.get_topic_info()

# Iterate through each topic, including topic -1
for index, row in topic_info.iterrows():
    topic = row.Topic
    freq  = row.Count  # Frequency of the topic
    # Retrieve the top keywords for the current topic (each element is (keyword, weight))
    keywords_with_weights = topic_model.get_topic(topic)

    # Show the top 10 keywords per topic
    top_10_keywords = keywords_with_weights[:10]

    # Extract just the keywords from the tuples
    keywords_only = [keyword for keyword, weight in top_10_keywords]

    print(f"Topic {topic} (Frequency: {freq}): {keywords_only}\n")
```

```
Topic -1 (Frequency: 959): ['hund', 'depression', 'reise']

Topic 0 (Frequency: 2206): ['sekundarschule', 'schule', 'klasse']

Topic 1 (Frequency: 673): ['beziehung', 'liebe', 'freundschaft']

Topic 2 (Frequency: 382): ['veränderung', 'persönlichkeit', 'gefühl']

Topic 3 (Frequency: 260): ['pandemie', 'grossmutter', 'bike']

Topic 4 (Frequency: 238): ['reise', 'ferien', 'auslandaufenthalt']

Topic 5 (Frequency: 235): ['familie', 'onkel', 'cousine']

Topic 6 (Frequency: 146): ['musik', 'band', 'geburtstagsesse']

Topic 7 (Frequency: 142): ['sport', 'PLACEHOLDER_PII', 'PLACEHOLDER_PII']

Topic 8 (Frequency: 128): ['verlobung', 'heirat', 'schwangerschaft']

Topic 9 (Frequency: 125): ['jobwechsel', 'berufswechsel', 'berufsfeldwechsel']

Topic 10 (Frequency: 109): ['elternhaus', 'wohngemeinschaft', 'wohngruppe']

Topic 11 (Frequency: 48): ['autoprüfung', 'auto', 'fahrprüfung']
```

In [11]:

```
# Download for MANUAL REVIEW
intial_TM = pd.DataFrame({"id": id, "age": age , "text": events, "topics": topics})

# Mapping dictionary: numbers -> topic labels
topic_mapping = {
    -1: "outlier",
    0: "School, Education, Apprenticeship",
    1: "Romantic Relationships & Friendships",
    2: "Mental Health, Development & Change",
    3: "Stressful Life Events & Loss",
    4: "Travelling, Vacation, Staying Abroad",
    5: "Family & Family-Related Events",
    6: "Celebrations & Going Out",
    7: "Sports",
```

```
    8: "Marriage & Parenthood",
    9: "Work, Employment",
    10: "Moving & Living on One’s Own",
    11: "Driver’s License & Vehicles"
}

# Recode the 'Topic' column to create a new 'Topic_Label' column:
intial_TM["topics"] = intial_TM["topics"].map(topic_mapping)


# Save the DataFrame to an Excel file
intial_TM.to_excel("/data/ALPACA_BERTopic/ALPACA_BERTopic_hard_clustering.xlsx", index=False)
```

In [18]:

```
####################################
#                                  #
#            TRAINING A            #
#      CLASSIFIER TO ALLOCATE      #
#           THE OUTLIERS           #
#                                  #
####################################



### The code concerning the training of the LLM-based classifier
### is based on the following Hugging Face Tutorial:
### https://huggingface.co/docs/transformers/training
```

In [85]:

```
# Load REVIEWED file (non-outliers only)
training_data   = pd.read_excel('/data/ALPACA_BERTopic/reviewed_BERTopic_training_data.xlsx', index_col=None, heade

### How many text segments were manually corrected?
# Count mismatches
num_mismatches = (training_data['topics_reviewed'] != training_data['topics_bertopic']).sum()
# Total number of rows
total_rows = len(training_data)
print(total_rows)
# Compute disagreement %
disagreement_percent = (num_mismatches / total_rows) * 100
# Show results
print(f"Text segments that were manually corrected: {disagreement_percent:.2f}%")
```

```
4706
Text segments that were manually corrected: 23.93%
```

In [86]:

```python
# Mapping dictionary: numbers -> topic labels
topic_mapping = {
      "School, Education, Apprenticeship": 0,
      "Romantic Relationships & Friendships": 1,
      "Mental Health, Development & Change": 2,
      "Stressful Life Events & Loss": 3,
      "Travelling, Vacation, Staying Abroad": 4,
      "Family & Family-Related Events": 5,
      "Celebrations & Going Out": 6,
      "Sports": 7,
      "Marriage & Parenthood": 8,
      "Work, Employment": 9,
      "Moving & Living on One’s Own": 10,
      "Driver’s License & Vehicles": 11,
      "outlier": 12   # -1 is not a possible value for classifier training
}

# Recode the 'Topic' column to create a new 'Topic_Label' column:
training_data['topics_reviewed_numeric'] = training_data['topics_reviewed'].map(topic_mapping)

# Check
print(training_data['topics_reviewed'].unique())
print(training_data['topics_reviewed_numeric'].unique())
```

```
['Romantic Relationships & Friendships'
 'School, Education, Apprenticeship' 'Driver’s License & Vehicles'
 'Mental Health, Development & Change' 'Sports'
 'Family & Family-Related Events' 'Travelling, Vacation, Staying Abroad'
 'Moving & Living on One’s Own' 'Work, Employment'
 'Celebrations & Going Out' 'Stressful Life Events & Loss' 'outlier'
 'Marriage & Parenthood']
[ 1  0 11  2  7  5  4 10  9  6  3 12  8]
```

In [87]:

```python
# Prepare data for model training

# Keep only the text and topics_reviewed columns
training_data = training_data[['text', 'topics_reviewed_numeric']]
```

```python
# Rename topics_reviewed to label
training_data = training_data.rename(columns={'topics_reviewed_numeric': 'label'})

# Check
print(training_data.columns)
```

```
Index(['text', 'label'], dtype='object')
```

In [40]:
```python
# Splitting the dataframe into training, and validation data
# while stratifying for topic categories

from sklearn.model_selection import train_test_split


train_df, val_df = train_test_split(
    training_data,
    test_size=0.30,
    stratify=training_data['label'],  # ensures even category distribution
    random_state=42                    # reproducibility
)
```

In [41]:
```python
# Import library
from datasets import Dataset

# Convert to Hugging Face dataset format
train = Dataset.from_pandas(train_df)
val   = Dataset.from_pandas(val_df)


# Tokenize data
from transformers import AutoTokenizer
tokenizer = AutoTokenizer.from_pretrained('/data/GermanBERTuncased')


def tokenize_function(examples):
    return tokenizer(examples["text"], padding="max_length", truncation=True)

train = train.map(tokenize_function, batched=True)
val   = val.map(tokenize_function, batched=True)
```

```
Map: 100%|██████████| 3294/3294 [00:00<00:00, 5547.55 examples/s]
Map: 100%|██████████| 1412/1412 [00:00<00:00, 6995.17 examples/s]
```

In [42]:

```python
# Load German BERT model (stored locally and loaded from disk)
# num_labels = needs to be set to the number of unique labels

from transformers import AutoModelForSequenceClassification

path = '/data/GermanBERTuncased'
model = AutoModelForSequenceClassification.from_pretrained(path, num_labels=13) # num_labels -> 12 Topics + outlier
```

```
Some weights of BertForSequenceClassification were not initialized from the model checkpoint at /data/chhaag/GermanB
ERTuncased and are newly initialized: ['classifier.bias', 'classifier.weight']
You should probably TRAIN this model on a down-stream task to be able to use it for predictions and inference.
```

In [43]:

```python
#  Training hyperparameters
from transformers import TrainingArguments, Trainer
import numpy as np
from sklearn.metrics import precision_recall_fscore_support, accuracy_score


def compute_metrics(eval_pred):
    logits, labels = eval_pred
    preds = np.argmax(logits, axis=-1)
    acc = accuracy_score(labels, preds)
    precision, recall, f1, _ = precision_recall_fscore_support(
        labels, preds, average='macro'
    )
    return {
        "eval_accuracy": acc,
        "eval_f1_macro": f1,
        "eval_precision_macro": precision,
        "eval_recall_macro": recall,
    }


# Monitoring evaluation metrics during fine-tuning
training_args = TrainingArguments(output_dir="/data/ALPACA/",
                                  eval_strategy="epoch",
                                  logging_dir='/data/ALPACA/',
```

```
                                  logging_steps=10,
                                  num_train_epochs=10,
                                  save_strategy="epoch",
                                  load_best_model_at_end=True,       # Load the best model based on evaluation metr
                                  metric_for_best_model="f1_macro",  # Metric to determine the best model
                                  greater_is_better=True  # For F1 score, higher is better
                                  )
```

In [44]:
```
# Prepare trainer
trainer = Trainer(model=model,
                  args=training_args,
                  train_dataset=train,
                  eval_dataset=val,
                  compute_metrics=compute_metrics)
```

In [45]:
```
# Run classifier training
trainer.train()
```

[4120/4120 06:04, Epoch 10/10]

Epoch	Training Loss	Validation Loss	Accuracy	F1 Macro	Precision Macro	Recall Macro
1	0.728800	0.411019	0.910765	0.800229	0.817388	0.800510
2	0.089100	0.332105	0.942635	0.859605	0.929859	0.850393
3	0.101200	0.368806	0.944051	0.857028	0.881255	0.854320
4	0.000700	0.358982	0.946176	0.875924	0.885690	0.873262
5	0.121600	0.397936	0.944759	0.884595	0.896003	0.879359
6	0.000700	0.441858	0.943343	0.872260	0.881303	0.873011
7	0.000500	0.432832	0.945467	0.874610	0.894893	0.863571
8	0.000300	0.428548	0.943343	0.870046	0.889059	0.861547
9	0.000300	0.436327	0.947592	0.874571	0.891635	0.867376
10	0.000300	0.437066	0.946176	0.874642	0.891094	0.867358

```
/data/chhaag/conda/envs/myenv3/lib/python3.11/site-packages/sklearn/metrics/_classification.py:1565: UndefinedMetric
Warning: Precision is ill-defined and being set to 0.0 in labels with no predicted samples. Use `zero_division` para
meter to control this behavior.
  _warn_prf(average, modifier, f"{metric.capitalize()} is", len(result))
```

Out[45]:
```
TrainOutput(global_step=4120, training_loss=0.09789968520692251, metrics={'train_runtime': 364.539, 'train_samples
_per_second': 90.361, 'train_steps_per_second': 11.302, 'total_flos': 8667734143979520.0, 'train_loss': 0.09789968
520692251, 'epoch': 10.0})
```

In [46]:
```python
# After training, the best model is automatically loaded and can be saved
model.save_pretrained("/data/ALPACA_BERTopic/best_topic_classification_model")
```

In [88]:
```python
import pandas as pd

#### Load BERTopic outliers which need to be assigned to topic
outliers   = pd.read_excel('/data/ALPACA_BERTopic/ALPACA_BERTopic_outliers.xlsx',
                           index_col=None, header=0)

print("Number of text segments flagged as outliers:", len(outliers))


# Load Transformer text classification pipeline
from transformers import pipeline

# Load fine-tuned model + its tokenizer
pipe = pipeline("text-classification",
                 model="/data/ALPACA_BERTopic/best_topic_classification_model/",
                 tokenizer="/data/ALPACA_BERTopic/best_topic_classification_model/",
                 device=0)

# Apply it to each row in the outliers df
outliers["predicted_topic_numeric"] = outliers["text"].apply(lambda txt: pipe(txt, truncation=True)[0]["label"])
```

```
Device set to use cpu
Number of text segments flagged as outliers: 964
```

In [3]:
```python
# Check df structure
print(outliers.loc[27:29, ['text', 'topics_bertopic', 'predicted_topic_numeric']])
```

```
                                text topics_bertopic predicted_topic_numeric
27  Ich habe eine Lehrstelle bekommen         outlier                 LABEL_0
28               Ich bekam eine Katze         outlier                 LABEL_5
29                          Beziehung         outlier                 LABEL_1
```

In [4]:

```
# Recode data
topic_mapping = {
      "LABEL_0": "School, Education, Apprenticeship",
      "LABEL_1": "Romantic Relationships & Friendships",
      "LABEL_2": "Mental Health, Development & Change",
      "LABEL_3": "Stressful Life Events & Loss",
      "LABEL_4": "Travelling, Vacation, Staying Abroad",
      "LABEL_5": "Family & Family-Related Events",
      "LABEL_6": "Celebrations & Going Out",
      "LABEL_7": "Sports",
      "LABEL_8": "Marriage & Parenthood",
      "LABEL_9": "Work, Employment",
      "LABEL_10": "Moving & Living on One’s Own",
      "LABEL_11": "Driver’s License & Vehicles",
      "LABEL_12": "outlier"
}

# Recode the 'Topic' column to create a new 'Topic_Label' column:
outliers["predicted_topic"] = outliers["predicted_topic_numeric"].map(topic_mapping)

print(outliers.loc[27:29, ['text', 'topics_bertopic', 'predicted_topic_numeric', 'predicted_topic']])

# Save the DataFrame to an Excel file for MANUAL REVIEW
outliers.to_excel("/data/ALPACA_BERTopic/ALPACA_classified_outliers.xlsx", index=False)
```

```
                                text topics_bertopic predicted_topic_numeric  \
27  Ich habe eine Lehrstelle bekommen         outlier                 LABEL_0
28               Ich bekam eine Katze         outlier                 LABEL_5
29                          Beziehung         outlier                 LABEL_1

                         predicted_topic
27     School, Education, Apprenticeship
28        Family & Family-Related Events
29  Romantic Relationships & Friendships
```

In [6]:
```
### After MANUAL REVIEW
#
#### Check manually reviewed outlier classifications
outliers_rev  = pd.read_excel('/data/ALPACA_BERTopic/ALPACA_classified_outliers_reviewed.xlsx',
                              index_col=None, header=0)

### How many text segments were manually corrected?
# Count mismatches
num_mismatches = (outliers_rev['predicted_topic'] != outliers_rev['topics_reviewed']).sum()
# Total number of rows
total_rows = len(outliers_rev)
# Compute disagreement %
disagreement_percent = (num_mismatches / total_rows) * 100
# Show results
print(f"Text segments that were manually corrected: {disagreement_percent:.2f}%")
```

```
Text segments that were manually corrected: 13.17%
```

In [2]:
```
####################################
#                                  #
#        CREATING THE FINAL        #
#       TOPIC REPRESENTATIONS      #
#                                  #
####################################

# Creating topics representations from the corrected topics
# Approach: Manual Topic Modelling - the topics from which we want to model the representations already exist
#
# The following code is based on this resource:
# https://maartengr.github.io/BERTopic/getting_started/manual/manual.html


import pandas as pd

# Load data
# Combine reviewed training data and outliers
training_data = pd.read_excel('/data/ALPACA_BERTopic/reviewed_BERTopic_training_data.xlsx',
                              index_col=None, header=0)

outliers_rev  = pd.read_excel('/data/ALPACA_BERTopic/ALPACA_classified_outliers_reviewed.xlsx',
```

```
                              index_col=None, header=0)

# Combine reviewed training data and outliers
bertopic_train = training_data [['text', 'topics_reviewed']]
outlier        = outliers_rev[[  'text', 'topics_reviewed']]

combined = pd.concat([bertopic_train, outlier], axis=0, ignore_index=True)

# Filter out the “outlier” rows
combined = combined[combined["topics_reviewed"] != "outlier"]


# Mapping dictionary: numbers -> topic labels
topic_mapping = {
      "School, Education, Apprenticeship": 0,
      "Romantic Relationships & Friendships": 1,
      "Mental Health, Development & Change": 2,
      "Stressful Life Events & Loss": 3,
      "Travelling, Vacation, Staying Abroad": 4,
      "Family & Family-Related Events": 5,
      "Celebrations & Going Out": 6,
      "Sports": 7,
      "Marriage & Parenthood": 8,
      "Work, Employment": 9,
      "Moving & Living on One’s Own": 10,
      "Driver’s License & Vehicles": 11
}

# Recode the 'Topic' column to create a new 'Topic_Label' column:
combined['topics_reviewed_numeric'] = combined['topics_reviewed'].map(topic_mapping)


# Check
print(combined["topics_reviewed"].unique())
print(combined["topics_reviewed_numeric"].unique())

# Head of the dataset
print(combined.loc[1:3,])
```

```
['Romantic Relationships & Friendships'
 'School, Education, Apprenticeship' 'Driver’s License & Vehicles'
 'Mental Health, Development & Change' 'Sports'
 'Family & Family-Related Events' 'Travelling, Vacation, Staying Abroad'
 'Moving & Living on One’s Own' 'Work, Employment'
 'Celebrations & Going Out' 'Stressful Life Events & Loss'
 'Marriage & Parenthood']
[ 1  0 11  2  7  5  4 10  9  6  3  8]
                                      text  \
1                          Mein Lehrbeginn
2  Das ich neue Freunde kennengelernt habe
3                        Meine Autoprüfung

                       topics_reviewed  topics_reviewed_numeric
1     School, Education, Apprenticeship                        0
2  Romantic Relationships & Friendships                        1
3           Driver’s License & Vehicles                       11
```

In [ ]:

```
### Creating empty instances of the dimensionality reduction and clustering steps
# see: https://maartengr.github.io/BERTopic/getting_started/manual/manual.html
from bertopic import BERTopic
from bertopic.backend import BaseEmbedder
from bertopic.cluster import BaseCluster
from bertopic.vectorizers import ClassTfidfTransformer
from bertopic.dimensionality import BaseDimensionalityReduction

### Prepare empty sub-models
empty_embedding_model = BaseEmbedder()
empty_dimensionality_model = BaseDimensionalityReduction()
empty_cluster_model = BaseCluster()
ctfidf_model = ClassTfidfTransformer(reduce_frequent_words=True)
```

In [5]:

```
### Lemmatization, part-of-speech tagging, stop word removal
### equivalent to original BERTopic model

import re
import spacy
import numpy as np
from nltk.corpus import stopwords
from sklearn.feature_extraction.text import CountVectorizer
```

```python
from bertopic.representation import MaximalMarginalRelevance
german_stop_words = stopwords.words('german')


# Expand stop words list and remove numbers
german_stop_words.extend(['jahr', 'monat', 'woche',
                          'januar', 'februar', 'märz', 'april', 'mai', 'juni', 'juli',
                          'august', 'september','oktober', 'november', 'dezember'])


# Lemmatizer
nlp = spacy.load("de_core_news_sm")
#
def lemma(text, allowed_postags=['NOUN']): # , 'ADJ','VERB','ADV'
    text = re.sub(r'\d+', '', text)
    text = re.sub(r'[,.!?+:-=`<>"/!()&%]', ' ', text)
    text = ' '.join([word.lemma_ for word in nlp(text) if word.pos_ in allowed_postags])
    text = text.lower()
    return text


vectorizer_model= CountVectorizer(stop_words=german_stop_words, preprocessor=lemma)
```

In [6]:

```python
# Prepare text data and topic labels for manual topic modelling
reviewed_labels = combined['topics_reviewed_numeric']
text = combined['text']
```

In [7]:

```python
# Fit BERTopic without actually performing any clustering
topic_model_reviewed= BERTopic(
        embedding_model=empty_embedding_model,
        umap_model=empty_dimensionality_model,
        hdbscan_model=empty_cluster_model,
        ctfidf_model=ctfidf_model,
        vectorizer_model=vectorizer_model
)

topics_reviewed, probs_reviewed = topic_model_reviewed.fit_transform(text, y=reviewed_labels)
```

In [81]:

```python
# Extract importance scores to create word clouds for visualization
```

```python
# Retrieve the topic info DataFrame
topic_info_reviewed = topic_model_reviewed.get_topic_info()

#
topic0 = {word: value for word, value in topic_model_reviewed.get_topic(0)}
topic1 = {word: value for word, value in topic_model_reviewed.get_topic(1)}
topic2 = {word: value for word, value in topic_model_reviewed.get_topic(2)}
topic3 = {word: value for word, value in topic_model_reviewed.get_topic(3)}
topic4 = {word: value for word, value in topic_model_reviewed.get_topic(4)}
topic5 = {word: value for word, value in topic_model_reviewed.get_topic(5)}
topic6 = {word: value for word, value in topic_model_reviewed.get_topic(6)}
topic7 = {word: value for word, value in topic_model_reviewed.get_topic(7)}
topic8 = {word: value for word, value in topic_model_reviewed.get_topic(8)}
topic9 = {word: value for word, value in topic_model_reviewed.get_topic(9)}
topic10 = {word: value for word, value in topic_model_reviewed.get_topic(10)}
topic11 = {word: value for word, value in topic_model_reviewed.get_topic(11)}
```

In [1]:
```python
# The key words were reviewed for identifiable content (replaced with [PLACEHOLDER]], then translated to English
# and again saved for word cloud creation

topic0 = {
    'high_school_diploma': 0.35171511388643767,
    'vocational_training': 0.3496955480161472,
    'studies': 0.34755905183903874,
    'school': 0.3402021116793455,
    'graduation': 0.3378090699591998,
    'grammar_school': 0.33706557976698387,
    'beginning': 0.3352568904514773,
    'secondary_school': 0.32846013206607294,
    'apprenticeship_position': 0.3282118400871645,
    'apprenticeship': 0.3274951591307959
}

topic1 = {
    'girlfriend': 0.6237219546754001,
    'relationship': 0.6195556328596504,
    'friend': 0.5955756026549298,
    'love': 0.5559181416328871,
    'friendship': 0.515942322912492,
    'person': 0.49734594563388823,
```

```
    'people': 0.4904953131299732,
    'meeting': 0.45292197922531197,
    'partner_(male)': 0.44209237896524983,
    'partner_(female)': 0.43317001957916457
}

topic2 = {
    'depression': 0.5802542120529912,
    'development': 0.5558634847421624,
    'life': 0.5174963837190061,
    'change': 0.502514888290813,
    'clinic': 0.49779438384425845,
    'self-discovery': 0.4656818531451606,
    'personality': 0.4656818531451606,
    'further_development': 0.44856513830014516,
    'band': 0.43567315737351264,
    'eating_disorder': 0.43567315737351264
}

topic3 = {
    'travel': 0.8584659107038285,
    'holidays': 0.8133475222201768,
    'language stay': 0.7364826884835372,
    'exchange year': 0.6528798976127536,
    'abroad': 0.6515901287766738,
    'gap_year': 0.6309814709181457,
    'summer_holidays': 0.6093907740588695,
    'stay_abroad': 0.5649015678132275,
    'visit_abroad': 0.5273766507913995,
    'month': 0.512099044682782
}

topic4 = {
    'job': 0.7527005698248783,
    'professional_life': 0.7058055546534882,
    'profession': 0.630479525893729,
    'work': 0.6207697580376733,
    'position': 0.619182751325489,
    'military': 0.5959138054209663,
    'job change': 0.5959138054209663,
    'workplace': 0.582871916621082,
```

```
    'career_choice': 0.5557465668200778,
    'professional_world': 0.5557465668200778
}

topic5 = {
    'family': 0.8003458262139544,
    'sister': 0.7218980523326822,
    'birth': 0.675677800729396,
    'brother': 0.6524396860640899,
    'parents': 0.6282156081208146,
    'uncle': 0.5627498641388194,
    'cousin': 0.5564495988112446,
    'father': 0.5427174696561817,
    'nephew': 0.5216296567978099,
    'cat': 0.514738476656023
}

topic6 = {
    'apartment': 0.9871520894722239,
    'moving_out': 0.9661608271775878,
    'home': 0.8790243174220415,
    'move': 0.8035470642184462,
    'house': 0.7839060979539398,
    'family_home': 0.7137999261519083,
    'parental_home': 0.6811336702481969,
    'move_out': 0.641651106785033,
    'shared_apartment': 0.5614903493463903,
    'shared_flat': 0.5614903493463903
}

topic7 = {
    'death': 1.0446323136326363,
    'grandfather': 0.7964828119443319,
    'grandmother': 0.7760573854654828,
    'pandemic': 0.7622995720575579,
    'mother': 0.754512174977677,
    'cancer': 0.6691988744204563,
    'breast_cancer': 0.6347021386012508,
    'illness': 0.6347021386012508,
    'father': 0.6227471115647009,
    'suicide_attempt': 0.5938017185950578
```

```
}

topic8 = {
    'sport': 0.9220193164176945,
    'football': 0.8943411513178037,
    'champion_(sport)': 0.713988447929085,
    'championship': 0.6771828407954734,
    'joining': 0.633544950003802,
    'athletics': 0.633544950003802,
    'national_team': 0.633544950003802,
    'handball': 0.633544950003802,
    'champion': 0.5798159830148145,
    'trial_session': 0.5798159830148145
}

topic9 = {
    'birthday': 1.6726453227859568,
    'concert': 1.1829323495969832,
    'justin': 1.13313551990892,
    'party': 0.8490034200802389,
    'festival_day': 0.7457839331894112,
    'christmas': 0.6932083896914609,
    '[PLACEHOLDER_LOCATION]': 0.591008437788823,
    'food': 0.591008437788823,
    'fan_meeting': 0.591008437788823,
    'graduation_ball': 0.591008437788823
}

topic10 = {
    'wedding': 1.2956548343261594,
    'engagement': 1.2439212304209395,
    'daughter': 1.184864931100459,
    'marriage': 1.1614299147735823,
    'son': 0.9272522127065305,
    'birth': 0.8963973533949292,
    'child': 0.7496997862841973,
    'pregnancy': 0.7076759402455468,
    'husband': 0.6070290647204242,
    'engaged': 0.6043872529647233
}
```

```
topic11 = {
    'driving test': 2.1113738621472824,
    'car': 1.58839331827611,
    'driving_exam': 1.346406558715645,
    'driving_license': 1.0126608950125626,
    'driving_permit': 0.8024993660183928,
    'dream_car': 0.8024993660183928,
    'moped': 0.8024993660183928,
    'motorcycle': 0.8024993660183928,
    'motorcycle_test': 0.8024993660183928,
    "driver's_license": 0.8024993660183928
}
```

In [ ]:

```
# Create word clouds

from wordcloud import WordCloud
import matplotlib.pyplot as plt

cloud = WordCloud(background_color='#D0F0F0',
                  # colors: teal: #D0F0F0; orange: #FFDAB933;
                  # purple #D6E7FF'; light blue #CCE7FF; dark blue: #D0E7FF
                  width=2500,
                  height=2200,
                  max_font_size=350,
                  relative_scaling=1.0,  # strict linear mapping
                  color_func=lambda *args, **kwargs: "black",
                  prefer_horizontal=1.0,
                  random_state=76)

# Generate the word cloud from frequencies
cloud.generate_from_frequencies(topic0)

plt.imshow(cloud, interpolation='bilinear')
plt.axis('off')
# Save the plot
plt.savefig('alp0.png', format='png', bbox_inches='tight', dpi=300)
```
