# Supplementary material for: Personally meaningful life events from adolescence to young adulthood: a longitudinal natural language processing analysis
Source: J Child Psychol Psychiatry. 2026 Jun 22;67(12):1937–52. doi: 10.1111/jcpp.70169 (PMC13745790; doi:10.1111/jcpp.70169)
Supplement: Supplementary file 3 [file JCPP-67-1937-s002.docx]

**Personally meaningful life events from adolescence to young adulthood: A longitudinal natural language processing analysis**

**Supporting Information**

**Table of Contents**

[Appendix S1. Complementary information of text pre-processing and the BERTopic analysis	2](#_Toc229383019)

[Appendix S2. Training an LLM-based classifier for emotional valence.	3](#_Toc229383020)

[Figure S1. Emotional Valence Classifier.	4](#_Toc229383021)

[Figure S2. Higher Order Topics Frequencies	5](#_Toc229383022)

[Figure S3. Life-Events Valence	5](#_Toc229383023)

[Figure S4. Life-Event Valence by Topic	6](#_Toc229383024)

[Figure S5. Life-Event Valence by Higher Order Topics	7](#_Toc229383025)

[Figure S6. Alluvial Plot of Higher Order Topics Transitions across Ages	8](#_Toc229383026)

[Figure S7. Associations between Symptoms of Anxiety/Depression with Life Event Topics	9](#_Toc229383027)

[Table S1. Topic Modelling Results: Topic Description and Text Examples	10](#_Toc229383028)

[Table S2. Logistic Regression Model Outputs (corresponding to Figure 4)	20](#_Toc229383029)

[Table S3. Logistic Regression Model Outputs Including Sociodemographic Factors and Age	24](#_Toc229383030)

[Table S4. Logistic Regression Model Outputs Including Sociodemographic Factors, Age and Internalizing Symptoms	30](#_Toc229383031)

[Table S5. Logistic Regression Model Outputs of Topic Valence by by Anxiety/Depression, SES, Sex, Migration Background, and Age.	36](#_Toc229383032)

# Appendix S1. Complementary information of text pre-processing and the BERTopic analysis

***Text data preprocessing.*** During spelling and punctuation corrections, we observed that participants used a mix of standard German expressions, Swiss-specific terminology, and official abbreviations when referring to different types of educational achievements. To minimize artificial differences, we automatically recoded all educational achievement terms into a uniform format before analysis (for full details, see the executed Jupyter notebook script ‘Appendix S3. ALPACA_BERTopic.pdf’). For subsequent analysis, we split up the text-data into the text segments based on periods and commas which was followed by a manual screening through LAB to ensure if this had resulted in text segments of similar length.

***English translation for visualization.*** The exemplary, anonymized quotes presented in the Table S1 were manually translated into English by LAB. Text data within the word clouds in Figure 1 were translated using GPT-4o and translations were manually reviewed by CH.

# Appendix S2. Training an LLM-based classifier for emotional valence.

Please find below a step-by-step description of training an LLM-based classifier to determine emotional valence of text-segments.

*Step 1 – ground truth data for model training*. The emotional valence of the life events at age 15 was assessed and coded by a rater as either positive, negative, or neutral based on face validity in August 2013 shortly after data assessment. The original rater was a psychologist (BSc) and supervised by the z-proso study PIs (ME, DR). Unclear entries were discussed and resolved among the study team. This data was later again manually reviewed by LAB and CH and corrected if needed (n=34, 0.02% corrected). All text data were used for classifier training described in the following, with no additional preprocessing steps applied.

*Step 2 – subdividing the ground truth data into training and validation datasets*. Again, we divided our data into training (80%) and validation subsets (20%), stratifying for the three emotional valence categories (positive, negative, ambivalent/ unclear).

*Step 3 – model training*. Equivalent to the topic classifier, we used the ‘Trainer’ class [42] from the Transformers Python library [11] for model training.

*Step 4 – classifier evaluation*. Equivalent to the topic classifier, we again focused on accuracy, F1 score, recall, and precision. For evaluation, we monitored metrics throughout training and selected the best-performing model based on the F1 score (for full details, see the executed Jupyter notebook script ‘Appendix S4. ALPACA_Emotional_Valence_Classifier.pdf’; the best model was trained in epoch 2, F1 = .850).

*Step 5 – Applying the classifier and manual review of the predicted classifications*. We then applied the classifier to text data assessed at ages 17, 20, and 24. Based on manual checks of the classification along the text narratives, we relabeled the neutral category into ‘ambivalent/unclear’ as many narratives either contained both positive and negative elements, while others were unclear in their valence but not clearly neutral (see Table S1, for topic descriptions and example narratives).To validate model performance all predictions checked manually by LAB and CH and were corrected if needed (12.61% of all predicted cases, with most corrections relating to the ‘ambivalent/ unclear’ category).

Figure S1. Emotional Valence Classifier. Visualization of Training an LLM-based classifier for emotional valence. Steps include: Step 1 – ground truth data for model training, Step 2 – subdividing the ground truth data into training and validation datasets, Step 3 – model training, Step 4 – classifier evaluation, & Step 5 – Applying the classifier and manual review of the predicted classifications. The executed Jupyter notebook script ‘Appendix S4. ALPACA_Emotional_Valence_Classifier.pdf’ is enclosed as further Appendix S4.

# Figure S2. Higher Order Topics Frequencies

**Figure S2.** Frequency plot of higher order life event topics displaying the frequency of the 5 higher order topics across all participants and measurement timepoints. The 5 topics are plotted on the y-axis, higher order topic frequency across participants (i.e., how many topics were mentioned within this higher-order category) is plotted on the x-axis.

# Figure S3. Life-Events Valence

**Figure S3.** Frequency plot of life event valence displaying the frequency of life-event valence across all participants and measurement timepoints. The valence categories are plotted on the x-axis, frequency across participants (i.e., how many topics were classified into the corresponding valence) is plotted on the y-axis.

# Figure S4. Life-Event Valence by Topic

**Figure S4.** Frequency plot of life-event valence by topic across all participants and measurement timepoints. The valence categories and topics are plotted on the x-axis, cell-percentage across all participants (i.e., how many participants topics were relatively classified into a given valence by topic) is plotted on the y-axis. Fisher's exact test was used to determine if the distribution of valences differs across topics (*N* = 5,620, df = 22, *Фс* =0.57, Fisher's p < 001). The low p-value (p < .001) indicates strong evidence against the null hypothesis, suggesting a significant association between the variables, thus, the distribution of valence varies depending on the topic.

# Figure S5. Life-Event Valence by Higher Order Topics

**Figure S5.** Frequency plot of life-event valence by higher-order topic across all participants and measurement timepoints. The valence categories and topics are plotted on the x-axis, cell-percentage across all participants (i.e., how many participants topics were relatively classified into a given valence by topic) is plotted on the y-axis. A chi-quare test was used to determine if the distribution of valences differs across higher-order topics (*N* = 5620, *χ^2^*= 1885.02, df = c(8), *Фс* = 0.41, p<.001,). The low p-value (p < .001) indicates strong evidence against the null hypothesis, suggesting a significant association between the variables, thus, the distribution of valence varies depending on the higher-order topic.

# Figure S6. Alluvial Plot of Higher Order Topics Transitions across Ages

**Figure S6.** Alluvial plots of higher-order life event topics and shifts in higher-order topics across measurement timepoints from adolescence (ages 15, 17) into young adulthood (ages 20, 24). Measurement timepoints/ages are plotted on the x-axis, frequency of higher-order topics is plotted on the y-axis. Topics are sorted along their overall frequency from most frequent higher-order topic (Education & Career Development) towards the least frequent higher-order topic (Other Life Transitions & Independence).

# Figure S7. Associations between Symptoms of Anxiety/Depression with Life Event Topics

**Figure S7.** Symptoms of anxiety/depression as predictor of life event topics, controlling for sex, SES, migration background and age. Estimates and corresponding 95%-confidence intervals result from 12 multivariable binomial logistic regressions modeling specific life event topics as a function of z-scaled dimensional anxiety/depression symptoms. Cluster-robust standard errors were used to control for clustering of data within individuals.

# Table S1. Topic Modelling Results: Topic Description and Text Examples

| **Topic** | **Summary** | **Exemplary event description and valence^a^** | | **Distribution of valence^ab^**  **N A/U P** | | |
| --- | --- | --- | --- | --- | --- | --- |
| **Education and career development** (n = 2873, 51.1%) | | | | n = 60  2.1% | n = 260  9% | n = 2553  88.9% |
| **School, education, and apprenticeship**  (n = 2504, 44.6%) | Numerous participants mentioned life events related to school, education, and apprenticeships. These were influenced by both positive achievements as well as certain challenges. Younger participants (ages 15-17) celebrated academic successes and securing apprenticeships, while also highlighting transitions between schools or to apprenticeships and some school-related struggles. Older participants (ages 20-24) reflected more on high school graduation, university life, and apprenticeship completion, with successes complemented by occasional setbacks, like dropping out of studies or dissolving contracts. Most events in this topic were related to transitions, such as graduations and starting an apprenticeship or university. While positive events and achievements generally outweighed negative events, struggles and challenging events were also reported across all age groups. | Age 15 | **School**   - *I passed the high school entrance exam. (P)* - *Good math grade. (P)* - *The years I was in boarding school. (A/U)* - *I had to repeat a grade. (N)* - *I always fought with a student. (N)* - *My unfair teacher gave me bad grades, so I did not pass high school’s last year. (N)*   **Apprenticeship (vocational training)**   - *When I signed my apprenticeship contract. (P)* - *When I got my internship position as a care professional (for children). (P)* | n = 54 2.2% | n = 186 7.4% | n = 2264 90.4% |
|  |  | Age 17 | **School**   - *Finishing secondary school. (P)* - *Entering high school. (P)* - *Changing schools. (A/U)* - *School dropout. (N)*   **University**   - *Going to university. (P)* - *Studies. (P)*   **Apprenticeship (vocational training)**   - *Starting my apprenticeship. (P)* - *Starting an internship. (P)* - *My apprenticeship contract was dissolved. (N)* |  |  |  |
|  |  | Age 20 | **School**   - *Leaving school and entering the real world. (P)* - *When I did not pass the high school exam and had to repeat the final year of high school. (N)*   **University**   - *Being accepted into medical school. (P)* - *A few years ago, I was abroad to study. (P)*   **Apprenticeship (vocational training)**   - *Finishing my apprenticeship. (P)* - *Dropping out of my apprenticeship (N)* |  |  |  |
|  |  | Age 24 | **School**   - *Finishing the vocational baccalaureate. (P)*   **University**   - *Bachelor graduation. (P)* - *Starting my studies abroad. (P)* - *Dropping out of my studies. (N)*   **Apprenticeship (vocational training)**   - *Finding an apprenticeship position. (P)* - *Finishing the apprenticeship. (P)* - *Starting my training in the medical field. (P)* |  |  |  |
| **Work and employment**  (n = 369, 6.6%) | Life events related to work and employment were reported across all age groups. Yet, this topic was less common among younger participants (ages 15-17), who mainly reported events related to career decisions and starting work. Older participants (ages 20-24) reflected more on career advancements like promotion, founding a business, and new positions. While many participants celebrated their entry into the workforce, some also experienced challenges as they navigated their careers. One subgroup of events in this topic was related to military service, which was mainly mentioned at ages 20 and 24. | Age 15 | - *Deciding what I will do in the future. (P)* - *Deciding what to work later in life. (P)* | n = 6 1.6% | n = 74 20.1% | n = 289 78.3% |
|  |  | Age 17 | - *Started to work. (P)* - *Starting work life. (P)* - *I found great work. (P)* |  |  |  |
|  |  | Age 20 | - *First work-related experiences. (P)* - *Unfortunately, I was often unemployed. (N)* - *Military, it changes you; you become an adult. (P)* - *Recruit school. (N)* |  |  |  |
|  |  | Age 24 | - *Got my first job. (P)* - *First work. (P)* - *Better job, higher salary. (P)* - *Establishing my own company. (P)* - *Promotion. (P)* - *My work in social services. (P)* - *Bad employer. (N)* - *The most important event was community service, which introduced me to another field. (P)* - *Finishing military service. (P)* |  |  |  |
| **Social relationships** (n = 1101, 19.6%) | | | | n = 79 7.2% | n = 53 4.8% | n = 969 88% |
| **Romantic relationships and friendships**  (n = 687, 12.2%) | Across all age groups, participants reported life events related to romantic relationships and friendships. Regarding relationship events, younger participants (ages 15-17) reported first relationships and sexual discoveries, while older participants (ages 20-24) described more complex dynamics such as long-term relationships and moving in with partners. Romantic relationships were mainly described as a source of joy, but break-ups emerged as negative experiences for some participants. Friendships were another key area of life events in this topic. Across all ages, participants valued meeting new friends and forming friendships. However, the loss of friends and interpersonal conflicts also had a notable impact, especially in younger participants. | Age 15 | **Romantic relationships and sexual events**   - *First girlfriend. (P)* - *My first time really being in love. (P)* - *I discovered my sexuality. (P)*   **Friendships**   - *I met new friends. (P)* - *My best friend moved away. (N)* - *Best friend disappeared out of life. (N)* | n = 38 5.5% | n = 28 4.1% | n = 621 90.4% |
|  |  | Age 17 | **Romantic relationships and sexual events**   - *My big love. (P)* - *First relationship. (P)* - *Split up from my violent boyfriend. (A/U)* - *My first kiss. (P)*   **Friendships**   - *I experienced a lot with my best friend. (P)* - *Switching friend groups. (A/U)* - *I protected a friend from her violent boyfriend. (A/U)* - *My best friend moved in with me. (P)* |  |  |  |
|  |  | Age 20 | **Romantic relationships and sexual events**   - *Starting a long-distance relationship. (P)* - *Meeting my fiancée. (P)* - *I had my first real and long relationship which shattered. (N)* - *First sexual experiences. (P)*   **Friendships**   - *Lost and found friends. (A/U)* |  |  |  |
|  |  | Age 24 | **Romantic relationships and sexual events**   - *First relationship. (P)* - *Finding my soulmate. (P)* - *Splitting up from my long-term boyfriend. (N)* - *Losing my virginity. (P)* - *Moving in with my boyfriend. (P)*   **Friendships**   - *I met a lot of new people. (P)* |  |  |  |
| **Family and family-related events** (n = 306, 5.4%) | Family and family-related events played a central role for participants across all ages. Memorable moments like reunions, births, and adopting pets often brought joy. However, stressful family dynamics, such as parental separations or mental health struggles, were also reported. As participants aged, their focus shifted more toward their independence from their birth family and managing complex family relationships, while the joys of welcoming new family members and pets and celebrating family-related events remained important. | Age 15 | **Family events, reunions, and newborns**   - *Better relationship with my mother. (P)* - *I saw my father again. (A/U)* - *My father remarried. (A/U)* - *I have a nephew. (P)* - *Seeing my mother pregnant. (P)*   **Stressful events**   - *My parents split up. (N)* - *My father’s drug addiction. (N)* - *My father’s psychosis. (N)*   **Pets/animals and other**   - *We bought a dog. (P)* - *I got a cat. (P)* - *My sister could handle her mental problems by moving out. (P)* - *I came into a foster family. (A/U)* | n = 37 12.1% | n = 24 7.8% | n = 245 80.1% |
|  |  | Age 17 | **Family events, reunions, and newborns**   - *My sister’s engagement. (P)* - *Meeting my uncle last year. (P)* - *My cousin’s baptism. (P)* - *Arrival of my family from abroad in Switzerland. (P)* - *I have another younger brother. (P)* - *My niece’s birth. (P)*   **Stressful events**   - *My parents had a court dispute. (N)* - *My mother left me and is not in touch. (N)*   **Pets/animals and other**   - *My pet “lives” with me. (P)* - *When I got my “own” pet. (P)* - *My parents are still alive. (P)* - *Sibling got out of jail. (A/U)* |  |  |  |
|  |  | Age 20 | **Family events, reunions, and newborns**   - *A few years ago, my mother returned from abroad, and we have been living together again since. (P)* - *My niece’s birth. (P)* - *And recently my godchild’s birth. (P)*   **Stressful events**   - *I haven’t seen my father since my parents’ break-up. (N)* - *Breaking off contact with family. (N)*   **Pets/animals and other**   - *Better relationship with my foster parents. (P)* |  |  |  |
|  |  | Age 24 | **Family events, reunions, and newborns**   - *My uncle and aunt adopted children. (P)*   **Stressful events**   - *My mother is increasingly doing worse mentally and is fully unstable. (N)* - *The maternal role I played for my brother and my mother destroyed me over time. (N)*   **Pets/animals and other**   - *I took over a small dog from a friend. (P)* - *Adopted a dog from the animal shelter. (P)* - *I became financially and generally independent from my father. (P)* |  |  |  |
| **Marriage and parenthood** (n = 108, 1.9%) | Marriage and parenthood were mentioned as important life events by numerous participants, often bringing joy and fulfillment. However, for some, these experiences also came with challenges, such as the pain of divorce or broken engagements. While reports of younger participants (ages 15-17) mainly involved family weddings, older participants (ages 20-24) frequently described experiencing their own marriage and parenthood. | Age 15 | - *I had extreme heartache. (N)* | n = 4 3.7% | n = 1 0.9% | n = 103 95.4% |
|  |  | Age 17 | - *My cousin’s wedding. (P)* - *My sister’s wedding. (P)* |  |  |  |
|  |  | Age 20 | - *I am married now. (P)* - *I had a religious wedding. (P)* - *I was proposed to. (P)* - *I became a mother. (P)* - *My son’s birth. (P)* |  |  |  |
|  |  | Age 24 | - *Engagement. (P)* - *Wedding. (P)* - *My husband could come to Switzerland, and we got married. (P)* - *The divorce of my marriage. (N)* - *Dissolution of engagement. (N)* - *In the meantime, I was lovesick, but shortly after I married my husband. (A/U)* - *My daughter’s birth. (P)* - *When I learnt about my pregnancy. (P)* |  |  |  |
| **Mental health and wellbeing** (n = 629, 11.2%) | | | | n = 211 33.5% | n = 42 6.7% | n = 376 589.8% |
| **Mental health, development, and change** (n = 443, 7.9%) | Participants across all age groups reported life events related to mental health, identity, and personal growth. Positive changes were reported, with some participants actively working on improving their well-being. Yet, some also mentioned mental health struggles such as self-harm or bullying. Reports of identity development were marked by self-acceptance and self-discovery (e.g., sexual orientation, spirituality). Life events related to physical changes and personality development contributed to a sense of change and personal growth. Religious events and experiences were mentioned in some accounts. Several participants also reported embracing various hobbies. As they matured, participants described greater responsibility and clarity in life, with mental health and development remaining a constant area of focus. | Age 15 | **Mental health**   - *Smoking my first joint at a water park. (P)* - *I stopped smoking weed. (A/U)* - *Eating disorder, anorexia. (N)*   **Identity, development, and change**   - *I started to come out (about one year ago). (P)* - *I got active in politics and enjoy standing up for a just world. (P)* - *I am more mature. (P)*   **Religion and other**   - *Changing my religion (atheist). (P)* - *My confirmation. (P)* - *Being accepted into theatre. (P)* | n = 46 10.4% | n = 36 8.1% | n = 361 81.5% |
|  |  | Age 17 | **Mental health**   - *Discovering I have ADD. (N)* - *Developing an obsessive-compulsive disorder. (N)*   **Identity, development, and change**   - *I am no longer afraid of being myself. (P)* - *Losing weight. (P)* - *Started playing am instrument. (P)* - *Got rid of my braces. (P)*   **Religion and other**   - *God’s vocation for me. (P)* - *I got a pet. (P)* |  |  |  |
|  |  | Age 20 | **Mental health**   - *Living better than a few years ago, meaning I no longer have suicidal thoughts. (P)* - *I got help to confront my intrusive memories to get rid of them. (P)* - *I fear it was when I realized that I considered self-harm and suicide as serious solutions. (N)*   **Identity, development, and change**   - *Entering the “[specific music style] scene”. (P)* - *I achieved a lot on the path to finding myself, concern myself more with spirituality, think more critically, and take my own path. (P)* - *I became more content with myself. (P)*   **Religion and other**   - *Learning about Buddhist values. (P)* - *The first performance with my band. (P)* - *Discovering nature, birds, animals, and trees. (P)* |  |  |  |
|  |  | Age 24 | **Mental health**   - *My alcohol abstinence. (P)* - *Bullying in one of my workplaces. (N)*   **Identity, development, and change**   - *Learning to deal with oneself. (P)* - *Having more responsibility in life. (P)*   **Religion and other**   - *The most important thing for me was my religious path. (P)* - *Abused trust. (N)* |  |  |  |
| **Stressful life events and loss** (n = 186, 3.3%) | Illness and health challenges, death and loss, and other stressful life events shaped participants' lives across all age groups. Illness, both own and that of loved ones, was recurrently reported as a struggle, with mixed outcomes of survival and ongoing battles with health. Loss was described as deeply impactful, with participants grieving family members, friends, and pets. Intense experiences such as rape and suicide attempts were also reported by some participants. At age 24, the COVID-19 pandemic and the war in Ukraine were mentioned by a number of participants, demonstrating the impact of the current world-political situation and societal challenges on young adults. | Age 15 | **Illness**   - *Big surgery. (N)* - *Broke my foot. (N)* - *My mother breast cancer. (P)* - *My mother has had breast cancer. (N)* - *I found a friend after their suicide attempt. (N)*   **Loss**   - *My close relative died, he was like my second father. (N)* - *My grandmother’s death. (N)* - *When my cat died, and I said goodbye to her. (N)* | n = 165 88.7% | n = 6 3.2% | n = 15 8.1% |
|  |  | Age 17 | **Illness**   - *Back surgery. (N)* - *Diagnosis of my illness. (N)* - *ACL tear. (N)* - *My mother’s suicide attempt. (N)* - *My mother developed cancer. (N)*   **Loss**   - *Death of a friend. (N)* - *My mother’s death. (N)*   **Other**   - *Saving a friend from a fight. (N)* - *Rape. (N)* - *Prison stay. (N)* |  |  |  |
|  |  | Age 20 | **Illness**   - *Accident with back problems. (N)* - *Survived a suicide attempt. (A/U)* - *My mother survived breast cancer. (P)* - *A relative wakes up from the coma. (P)*   **Loss**   - *My father’s death. (N)* - *My best friend’s death. (N)* |  |  |  |
|  |  | Age 24 | **Illness**   - *Illness caused by a pain syndrom. (N)*   **Loss**   - *My sister died. (N)* - *My best friend took her own life. (N)*   **World events**   - *Effects of the pandemic, division of society. (N)* - *World political events, Corona, Ukraine war, etc. (N)* - *War in Ukraine. (N)* |  |  |  |
| **Leisure activities and successes** (n = 697, 12.4%) | | | | n = 4 0.6% | n = 43 6.2% | n = 650 93.3% |
| **Travelling, vacation, and staying abroad** (n = 415, 7.4%) | Travel, vacations, and stays abroad were reported as important life events by participants of all age groups. Younger participants (age 15) mainly mentioned family holidays, while older age was increasingly associated with accounts of independent travels, personally meaningful trips, and cultural experiences. While language stays were mentioned across all age groups, they were less frequent among younger participants (age 15) and more common in older participants, with the duration of these stays gradually increasing to longer experiences abroad, gap years, and exchange semesters. Another subgroup of life events in this topic involved moving abroad, which was reported across all age groups and increasingly so at ages 20 and 24. While most of the life events in this topic were positive, a few experiences highlight the challenges associated with travels and staying abroad. | Age 15 | **Travelling, vacation**   - *Holidays abroad with parents. (P)* - *The cruise. (P)* - *A vacation week with my friend. (P)* - *When I was in America, my family and I got into a shooting. (N)*   **Staying abroad, exchange**   - *My language stay. (P)*   **Moving abroad**   - *Moving to Southern Europe. (N)* - *Moving to North Africa. (N)* | n = 2 0.5% | n = 28 6.7% | n = 385 92.8% |
|  |  | Age 17 | **Travelling, vacation**   - *Travelling to the middle east. (P)* - *School trip abroad with my class. (P)* - *First vacation abroad without parents. (P)*   **Staying abroad, exchange**   - *Language stay abroad. (P)* - *I was on exchange in northern Europe. (P)* |  |  |  |
|  |  | Age 20 | **Travelling, vacation**   - *High school graduation trip. (P)*   **Staying abroad, exchange**   - *Gap year with work experience and travelling. (P)* - *My volunteer work abroad. (P)*   **Moving abroad**   - *I moved to Latin America (A/U)* - *Moved to another country with my boyfriend. (A/U)* |  |  |  |
|  |  | Age 24 | **Travelling, vacation**   - *Going on a world trip. (P)* - *My long-distance hike. (P)*   **Staying abroad, exchange**   - *Language stay in the pacific region. (P)* - *Exchange year in southern Europe (P)* - *Internships abroad during my studies. (P)* - *Terminating my stays abroad. (N)*   **Moving abroad**   - *I moved to southern Africa. (A/U)* - *Moving to an urban metropole. (N)* |  |  |  |
| **Sports** (n = 174, 3.1%) | Sports played a central role in the lives of participants across all ages. Younger participants (ages 15-17) also described sports events experienced as fans. Personal sports achievements were recalled across all age groups, including some reports of professional or competitive sports experiences. Despite certain setbacks, the overall tone of the life event descriptions in this topic remained positive. | Age 15 | - *Training in a boxing club. (P)* - *Competing in the national championship. (P)* - *Being on the national team. (P)* - *Participating in the junior world championship. (P)* - *Barcelona’s 4:0 victory against Milan. (P)* | n = 2 1.1% | n = 12 6.9% | n = 160 92% |
|  |  | Age 17 | - *2^nd^ place in handball. (P)* - *[soccer club] wanted me. (P)* - *Saw the national team. (P)* - *Had to stop karate. (N)* |  |  |  |
|  |  | Age 20 | - *I started dancing. (P)* - *I skydived. (P)* |  |  |  |
|  |  | Age 24 | - *Getting closer to the world of e-sport. (P)* - *National champion. (P)* - *Qualification Olympics. (P)* |  |  |  |
| **Celebrations and going out** (n = 108, 1.9%) | Celebrations and concerts provided participants with moments of joy and excitement across all age groups. Concerts and celebrity interactions were described as unforgettable experiences. At age 15, participants repeatedly mentioned the Jusin Bieber concert, but other musical experiences were also recalled across the age groups. By age 24, concert mentions faded slightly. Other celebrations were still fondly remembered, and birthdays, holidays, and local festivals remained important. | Age 15 | - *I celebrated my birthday. (P)* - *The New Year’s Eve party 2012-2013. (P)* - *[specific Zurich-based holiday]. (A/U)* - *[specific festival] in summer 2012. (P)* - *Touching Justin Bieber. (P)* - *When I organized a big fan event on YouTube. (P)* - *Chris Brown concert. (P)* | n = 0 0% | n = 3 2.8% | n = 105 97.2% |
|  |  | Age 17 | - *Birthday dinner on my 18th birthday. (P)* - *Prom. (P)* - *Celebrating the holidays with my parents. (P)* - *ASAP Rocky concert. (P)* - *[large] open-air. (P)* - *Going on a worldwide tour as an engineer. (P)* - *Lady Gaga concert. (P)* |  |  |  |
|  |  | Age 20 | - *Finally turning 21. (P)* - *Festival in south eastern Europe. (P)* |  |  |  |
|  |  | Age 24 | - *Being able to celebrate my birthday before Corona happened. (P)* - *I liked the summer most; I think it was the Zurich festival. (P)* |  |  |  |
| **Other life transitions and independence** (n = 320, 5.7%) | | | | n = 1 0.3% | n = 199 62.2% | n = 120 37.5% |
| **Moving and living on one’s own**  (n = 263, 4.7%) | Changes in the living situation played a major role in the lives of participants, with both positive and challenging aspects mentioned. Younger participants (ages 15-17) often described changes in living situations tied to the family’s living condition or boarding school. Older participants (ages 20-24) frequently reported moving into their first flat and/or moving in with a partner. While most events in this topic were classified as neutral or positive, some can also be considered negative. | Age 15 | - *Boarding school. (P)* - *We finally have a flat that is big enough for all. (P)* - *Moving out of the children’s home. (A/U)* - *I moved from back to Switzerland. (A/U)* - *Moving in with my biological mother. (P)* | n = 1 0.4% | n = 199 75.7% | n = 63 24% |
|  |  | Age 17 | - *Entering boarding school. (P)* - *Feeling good in the new flat. (P)* - *Time in secure residential group. (A/U)* - *Moving out of home. (A/U)* - *Changing children’s home. (A/U)* |  |  |  |
|  |  | Age 20 | - *Moving out of my mother’s, basically the step into complete independence. (P)* - *I moved from home into a shared flat. (A/U)* - *First own flat. (A/U)* |  |  |  |
|  |  | Age 24 | - *Own flat. (P)* - *Moving into our own flat with my boyfriend. (P)* - *Moving from home into the shared flat. (A/U)* - *I came back to Switzerland. (A/U)* |  |  |  |
| **Driver’s license and vehicles** (n = 57, 1.0%) | For several participants, earning their driver’s license and purchasing a vehicle represented important life events. These were consistently positive. While mobility was mentioned across all age groups, related life events were mainly reported from age 17 onwards. | Age 15 | - *I have my own moped. (P)* - *Having the moped license. (P)* | n = 0 0% | n = 0 0% | n = 57 100% |
|  |  | *Age 17* | - *My driver’s license. (P)* - *Passing the motorcycle test. (P)* - *Driving test. (P)* |  |  |  |
|  |  | *Age 20* | - *Driving test and bought a BMW. (P)* - *Bought a car. (P)* - *Passed the driving test. (P)* - *New car. (P)* |  |  |  |
|  |  | *Age 24* | - *The first own car. (P)* - *My driver’s license. (P)* - *Driving a car. (P)* |  |  |  |
| **Total number of entries (excl. outliers) N = 5,620^c^** | | | | **n = 355 6.3%** | **n = 597** **10.6%** | **n = 4668** **83.1%** |
| **Outliers: Health and other micro-topics** (n = 50, 0.9%) | Some other life events were either reported only by a few participants or could not be readily assigned to a topic due to a lack of specificity. Among these, personal health was reported by participants across all age groups. Participants expressed their gratitude for being healthy and their hope to maintain their health. Some other micro-topics include unique experiences (e.g., winning money, helicopter flight) and youth group-related events and achievements (e.g., scouts, youth orchestra). | *Age 15* | - *That I am healthy. (P)* - *I received the Swiss passport. (P)* - *When I won 300 Euros. (P)* - *Helicopter flight with my mother. (P)* - *Became scout leader. (P)* |  |  |  |
|  |  | *Age 17* | - *I am glad to be healthy. (P)* - *I became a scout leader. (P)* - *Receiving the leading role in the school musical. (P)* |  |  |  |
|  |  | *Age 20* | - *That I stay healthy. (P)* - *My health. (P)* - *Participating in the world music festival with my youth orchestra, I played a lot of concerts. (P)* |  |  |  |
|  |  | *Age 24* | - *My health. (P)* - *Being happy and healthy. (P)* - *I left society/civilization. (A/U)* - *The whole court process. (N)* |  |  |  |
| **Total number of entries (including outliers) N = 5,670^d^** | | | | | | |

Note. ^a^ The valence was determined by the coding algorithm. N = negative, A/U = ambivalent/unclear, P = positive; ^b^ The percentages constitute row percentages, i.e., the distribution of valence within one topic; ^c^ N = 5,620 is the reference for calculating the percentages of all life-event topics; ^d^ N = 5670 is the reference for calculating the percentages of the outlier category. The exemplary, anonymized quotes presented in the Table S1 were manually translated into English by LAB. DB and CH performed further additional anonymization of quotes and changed translated text segments to make them less detailed and more general examples.

# Table S2. Logistic Regression Model Outputs (corresponding to Figure 4)

a) **Logistic Regression predicting the Topic ‘School, Education, & Apprenticeship’ by SES, Sex, and Migration Background.**

| Parameter | Odds Ratio | SE | 95%-CI  low | 95%-CI  high | z | p |
| --- | --- | --- | --- | --- | --- | --- |
| (Intercept) | 1.101 | 0.065 | 0.981 | 1.235 | 1.631 | .103 |
| SES | 0.988 | 0.035 | 0.921 | 1.059 | -0.355 | .723 |
| Sex (Wom.) | 0.975 | 0.064 | 0.857 | 1.108 | -0.394 | .693 |
| Mig. | 1.016 | 0.074 | 0.881 | 1.171 | 0.214 | .830 |

Tjur's R2 Values < 0.001

b) **Logistic Regression predicting the Topic ‘Romantic Relationships & Friendship’ by SES, Sex, and Migration Background.**

| Parameter | Odds Ratio | SE | 95%-CI  low | 95%-CI  high | z | p |
| --- | --- | --- | --- | --- | --- | --- |
| (Intercept) | 0.174 | 0.014 | 0.148 | 0.204 | -21.302 | <.001 |
| SES | 1.033 | 0.054 | 0.933 | 1.143 | 0.617 | .537 |
| Sex (Wom.) | 0.952 | 0.088 | 0.794 | 1.142 | -0.526 | .599 |
| Mig. | 0.959 | 0.098 | 0.785 | 1.172 | -0.408 | .683 |

Tjur's R2 Values = 0.002

c) **Logistic Regression predicting the Topic ‘Mental Health, Development, & Change’ by SES, Sex, and Migration Background.**

| Parameter | Odds Ratio | SE | 95%-CI  low | 95%-CI  high | z | p |
| --- | --- | --- | --- | --- | --- | --- |
| (Intercept) | 0.086 | 0.009 | 0.070 | 0.107 | -22.619 | <.001 |
| SES | 1.069 | 0.066 | 0.948 | 1.206 | 1.090 | .276 |
| Sex (Wom.) | 0.933 | 0.111 | 0.739 | 1.178 | -0.584 | .559 |
| Mig. | 1.240 | 0.164 | 0.957 | 1.607 | 1.627 | .104 |

Tjur's R2 Values = 0.001

d) **Logistic Regression predicting the Topic ‘Travelling, Vacation, & Staying Abroad’ by SES, Sex, and Migration Background.**

| Parameter | Odds Ratio | SE | 95%-CI  low | 95%-CI  high | z | p |
| --- | --- | --- | --- | --- | --- | --- |
| (Intercept) | 0.080 | 0.008 | 0.066 | 0.099 | -24.147 | <.001 |
| SES | **1.419** | 0.097 | 1.241 | 1.623 | 5.104 | **<.001** |
| Sex (Wom.) | **1.409** | 0.163 | 1.123 | 1.766 | 2.967 | **.003** |
| Mig. | 0.821 | 0.111 | 0.629 | 1.071 | -1.453 | .146 |

Tjur's R2 Values = 0.015

e) **Logistic Regression predicting the Topic ‘Work & Employment’ by SES, Sex, and Migration Background.**

| Parameter | Odds Ratio | SE | 95%-CI  low | 95%-CI  high | z | p |
| --- | --- | --- | --- | --- | --- | --- |
| (Intercept) | 0.093 | 0.009 | 0.076 | 0.113 | -23.538 | <.001 |
| SES | **0.822** | 0.053 | 0.725 | 0.933 | -3.025 | **.002** |
| Sex (Wom.) | 0.821 | 0.095 | 0.655 | 1.030 | -1.708 | .088 |
| Mig. | 0.935 | 0.125 | 0.719 | 1.216 | -0.502 | .616 |

Tjur's R2 Values = 0.003

f) **Logistic Regression predicting the Topic ‘Family & family-related Events’ by SES, Sex, and Migration Background.**

| Parameter | Odds Ratio | SE | 95%-CI  low | 95%-CI  high | z | p |
| --- | --- | --- | --- | --- | --- | --- |
| (Intercept) | 0.042 | 0.006 | 0.032 | 0.055 | -23.322 | <.001 |
| SES | **0.860** | 0.061 | 0.749 | 0.989 | -2.117 | **.034** |
| Sex (Wom.) | **1.751** | 0.240 | 1.338 | 2.290 | 4.086 | **<.001** |
| Mig. | **1.348** | 0.196 | 1.014 | 1.792 | 2.058 | **.040** |

Tjur's R2 Values = 0.009

g) **Logistic Regression predicting the Topic ‘Moving & Living on One's Own’ by SES, Sex, and Migration Background.**

| Parameter | Odds Ratio | SE | 95%-CI  low | 95%-CI  high | z | p |
| --- | --- | --- | --- | --- | --- | --- |
| (Intercept) | 0.047 | 0.006 | 0.036 | 0.061 | -22.320 | <.001 |
| SES | 0.943 | 0.075 | 0.807 | 1.102 | -0.734 | .463 |
| Sex (Wom.) | **2.137** | 0.314 | 1.602 | 2.851 | 5.166 | **<.001** |
| Mig. | **0.617** | 0.106 | 0.441 | 0.864 | -2.807 | **.005** |

Tjur's R2 Values = 0.010

h) **Logistic Regression predicting the Topic ‘Stressful Life Events & Loss’ by SES, Sex, and Migration Background.**

| Parameter | Odds Ratio | SE | 95%-CI  low | 95%-CI  high | z | p |
| --- | --- | --- | --- | --- | --- | --- |
| (Intercept) | 0.034 | 0.006 | 0.025 | 0.048 | -19.856 | <.001 |
| SES | 1.150 | 0.113 | 0.949 | 1.393 | 1.424 | .154 |
| Sex (Wom.) | **1.592** | 0.270 | 1.142 | 2.221 | 2.742 | **.006** |
| Mig. | 0.749 | 0.145 | 0.513 | 1.094 | -1.495 | .135 |

Tjur's R2 Values = 0.004

i) **Logistic Regression predicting the Topic ‘Sports’ by SES, Sex, and Migration Background.**

| Parameter | Odds Ratio | SE | 95%-CI  low | 95%-CI  high | z | p |
| --- | --- | --- | --- | --- | --- | --- |
| (Intercept) | 0.069 | 0.009 | 0.053 | 0.089 | -20.287 | <.001 |
| SES | 0.954 | 0.099 | 0.777 | 1.170 | -0.456 | .648 |
| Sex (Wom.) | **0.248** | 0.059 | 0.155 | 0.396 | -5.833 | **<.001** |
| Mig. | 0.713 | 0.153 | 0.469 | 1.085 | -1.580 | .114 |

Tjur's R2 Values = 0.013

j) **Logistic Regression predicting the Topic ‘Marriage & Parenthood’ by SES, Sex, and Migration Background.**

| Parameter | Odds Ratio | SE | 95%-CI  low | 95%-CI  high | z | p |
| --- | --- | --- | --- | --- | --- | --- |
| (Intercept) | 0.010 | 0.002 | 0.006 | 0.015 | -20.683 | <.001 |
| SES | **0.789** | 0.088 | 0.635 | 0.982 | -2.126 | **.033** |
| Sex (Wom.) | **1.990** | 0.447 | 1.281 | 3.090 | 3.065 | **.002** |
| Mig. | **2.108** | 0.505 | 1.318 | 3.372 | 3.111 | **.002** |

Tjur's R2 Values = 0.009

k) **Logistic Regression predicting the Topic ‘Celebrations & Going out’ by SES, Sex, and Migration Background.**

| Parameter | Odds Ratio | SE | 95%-CI  low | 95%-CI  high | z | p |
| --- | --- | --- | --- | --- | --- | --- |
| (Intercept) | 0.022 | 0.004 | 0.015 | 0.031 | -20.360 | <.001 |
| SES | 1.059 | 0.106 | 0.870 | 1.289 | 0.569 | .569 |
| Sex (Wom.) | 0.959 | 0.200 | 0.638 | 1.442 | -0.201 | .841 |
| Mig. | 1.242 | 0.273 | 0.808 | 1.909 | 0.986 | .324 |

Tjur's R2 Values < 0.001

l) **Logistic Regression predicting the Topic ‘Driver's License & Vehicles’ by SES, Sex, and Migration Background.**

| Parameter | Odds Ratio | SE | 95%-CI  low | 95%-CI  high | z | p |
| --- | --- | --- | --- | --- | --- | --- |
| (Intercept) | 0.009 | 0.003 | 0.005 | 0.016 | -15.395 | <.001 |
| SES | **0.477** | 0.086 | 0.335 | 0.678 | -4.122 | **<.001** |
| Sex (Wom.) | **0.412** | 0.132 | 0.220 | 0.773 | -2.760 | **.006** |
| Mig. | 1.723 | 0.607 | 0.863 | 3.438 | 1.542 | .123 |

Tjur's R2 Values = 0.011

# Table S3. Logistic Regression Model Outputs Including Sociodemographic Factors and Age

a) **Logistic Regression predicting the Topic ‘School, Education, & Apprenticeship’ by SES, Sex, Migration Background, and Age.**

| Parameter | Odds Ratio | SE | 95%-CI  low | 95%-CI  high | z | p |
| --- | --- | --- | --- | --- | --- | --- |
| (Intercept) | 0.863 | 0.067 | 0.742 | 1.005 | -1.894 | .058 |
| SES | 0.987 | 0.036 | 0.918 | 1.061 | -0.353 | .724 |
| Sex (Wom.) | 0.967 | 0.066 | 0.847 | 1.104 | -0.498 | .619 |
| Mig. | 1.014 | 0.077 | 0.874 | 1.176 | 0.182 | .856 |
| *Ref. Age 15* |  |  |  |  |  |  |
| Age 17 | **1.343** | 0.106 | 1.151 | 1.567 | 3.751 | **<.001** |
| Age 20 | **2.430** | 0.207 | 2.056 | 2.872 | 10.416 | **<.001** |
| Age 24 | 0.867 | 0.073 | 0.736 | 1.022 | -1.700 | .089 |

Tjur’s R2 Values = 0.036

b) **Logistic Regression predicting the Topic ‘Romantic Relationships & Friendship’ by SES, Sex, Migration Background, and Age.**

| Parameter | Odds Ratio | SE | 95%-CI  low | 95%-CI  high | z | p |
| --- | --- | --- | --- | --- | --- | --- |
| (Intercept) | 0.194 | 0.020 | 0.159 | 0.237 | -16.027 | <.001 |
| SES | 1.032 | 0.054 | 0.932 | 1.143 | 0.606 | .545 |
| Sex (Wom.) | 0.954 | 0.089 | 0.795 | 1.145 | -0.501 | .616 |
| Mig. | 0.959 | 0.098 | 0.785 | 1.173 | -0.404 | .686 |
| *Ref. Age 15* |  |  |  |  |  |  |
| Age 17 | 0.821 | 0.094 | 0.655 | 1.028 | -1.720 | .086 |
| Age 20 | **0.742** | 0.090 | 0.585 | 0.941 | -2.461 | **.014** |
| Age 24 | 1.007 | 0.116 | 0.803 | 1.263 | 0.061 | .952 |

Tjur's R2 Values = 0.002

c) **Logistic Regression predicting the Topic ‘Mental Health, Development, & Change’ by SES, Sex, Migration Background, and Age.**

| Parameter | Odds Ratio | SE | 95%-CI  low | 95%-CI  high | z | p |
| --- | --- | --- | --- | --- | --- | --- |
| (Intercept) | 0.085 | 0.012 | 0.065 | 0.112 | -17.763 | <.001 |
| SES | 1.061 | 0.066 | 0.940 | 1.198 | 0.962 | .336 |
| Sex (Wom.) | 0.930 | 0.111 | 0.736 | 1.176 | -0.605 | .545 |
| Mig. | 1.247 | 0.166 | 0.961 | 1.619 | 1.658 | .097 |
| *Ref. Age 15* |  |  |  |  |  |  |
| Age 17 | **0.709** | 0.108 | 0.525 | 0.957 | -2.246 | **.025** |
| Age 20 | 0.912 | 0.138 | 0.677 | 1.227 | -0.610 | .542 |
| Age 24 | **1.467** | 0.203 | 1.119 | 1.923 | 2.772 | **.006** |

Tjur's R2 Values = 0.006

d) **Logistic Regression predicting the Topic ‘Travelling, Vacation, & Staying Abroad’ by SES, Sex, Migration Background, and Age.**

| Parameter | Odds Ratio | SE | 95%-CI  low | 95%-CI  high | z | p |
| --- | --- | --- | --- | --- | --- | --- |
| (Intercept) | 0.079 | 0.011 | 0.060 | 0.103 | -18.215 | <.001 |
| SES | 1.423 | 0.098 | 1.243 | 1.629 | 5.115 | **<.001** |
| Sex (Wom.) | 1.409 | 0.163 | 1.124 | 1.768 | 2.970 | **.003** |
| Mig. | 0.820 | 0.111 | 0.628 | 1.070 | -1.464 | .143 |
| *Ref. Age 15* |  |  |  |  |  |  |
| Age 17 | 1.139 | 0.160 | 0.866 | 1.500 | 0.930 | .352 |
| Age 20 | 1.101 | 0.162 | 0.826 | 1.469 | 0.657 | .511 |
| Age 24 | 0.856 | 0.136 | 0.628 | 1.168 | -0.980 | .327 |

Tjur's R2 Values = 0.017

e) **Logistic Regression predicting the Topic ‘Work & Employment’ by SES, Sex, Migration Background, and Age.**

| Parameter | Odds Ratio | SE | 95%-CI  low | 95%-CI  high | z | p |
| --- | --- | --- | --- | --- | --- | --- |
| (Intercept) | 0.015 | 0.004 | 0.009 | 0.026 | -15.236 | <.001 |
| SES | **0.788** | 0.054 | 0.690 | 0.901 | -3.482 | **<.001** |
| Sex (Wom.) | 0.793 | 0.097 | 0.625 | 1.007 | -1.899 | .058 |
| Mig. | 0.956 | 0.135 | 0.725 | 1.262 | -0.316 | .752 |
| *Ref. Age 15* |  |  |  |  |  |  |
| Age 17 | **3.099** | 0.913 | 1.740 | 5.520 | 3.840 | **<.001** |
| Age 20 | **6.802** | 1.853 | 3.988 | 11.601 | 7.039 | **<.001** |
| Age 24 | **16.922** | 4.382 | 10.187 | 28.109 | 10.924 | **<.001** |

Tjur's R2 Values = 0.063

f) **Logistic Regression predicting the Topic ‘Family & family-related Events’ by SES, Sex, Migration Background, and Age.**

| Parameter | Odds Ratio | SE | 95%-CI  low | 95%-CI  high | z | p |
| --- | --- | --- | --- | --- | --- | --- |
| (Intercept) | 0.063 | 0.010 | 0.047 | 0.086 | -18.044 | <.001 |
| SES | **0.866** | 0.062 | 0.752 | 0.997 | -2.003 | **.045** |
| Sex (Wom.) | **1.775** | 0.245 | 1.354 | 2.328 | 4.150 | **<.001** |
| Mig. | **1.340** | 0.196 | 1.006 | 1.785 | 2.002 | **.045** |
| *Ref. Age 15* |  |  |  |  |  |  |
| Age 17 | **0.664** | 0.101 | 0.494 | 0.894 | -2.696 | **.007** |
| Age 20 | **0.462** | 0.084 | 0.324 | 0.658 | -4.271 | **<.001** |
| Age 24 | **0.484** | 0.087 | 0.340 | 0.687 | -4.053 | **<.001** |

Tjur's R2 Values = 0.016

g) **Logistic Regression predicting the Topic ‘Moving & Living on One's Own’ by SES, Sex, Migration Background, and Age.**

| Parameter | Odds Ratio | SE | 95%-CI  low | 95%-CI  high | z | p |
| --- | --- | --- | --- | --- | --- | --- |
| (Intercept) | 0.022 | 0.005 | 0.015 | 0.033 | -18.359 | <.001 |
| SES | 0.914 | 0.076 | 0.777 | 1.075 | -1.084 | .278 |
| Sex (Wom.) | **2.177** | 0.332 | 1.615 | 2.935 | 5.104 | **<.001** |
| Mig. | **0.616** | 0.110 | 0.433 | 0.874 | -2.713 | **.007** |
| *Ref. Age 15* |  |  |  |  |  |  |
| Age 17 | 0.700 | 0.186 | 0.416 | 1.177 | -1.345 | .179 |
| Age 20 | **1.699** | 0.373 | 1.105 | 2.614 | 2.414 | **.016** |
| Age 24 | **5.672** | 1.153 | 3.808 | 8.450 | 8.535 | **<.001** |

Tjur's R2 Values = 0.055

h) **Logistic Regression predicting the Topic ‘Stressful Life Events & Loss’ by SES, Sex, Migration Background, and Age.**

| Parameter | Odds Ratio | SE | 95%-CI  low | 95%-CI  high | z | p |
| --- | --- | --- | --- | --- | --- | --- |
| (Intercept) | 0.044 | 0.009 | 0.030 | 0.064 | -16.126 | <.001 |
| SES | 1.149 | 0.114 | 0.947 | 1.395 | 1.405 | .160 |
| Sex (Wom.) | **1.607** | 0.275 | 1.150 | 2.247 | 2.777 | **.005** |
| Mig. | 0.748 | 0.146 | 0.511 | 1.096 | -1.487 | .137 |
| *Ref. Age 15* |  |  |  |  |  |  |
| Age 17 | **0.671** | 0.135 | 0.452 | 0.995 | -1.984 | **.047** |
| Age 20 | **0.329** | 0.090 | 0.192 | 0.562 | -4.068 | **<.001** |
| Age 24 | 1.114 | 0.214 | 0.764 | 1.623 | 0.560 | .576 |

Tjur's R2 Values = 0.011

i) **Logistic Regression predicting the Topic ‘Sports’ by SES, Sex, Migration Background, and Age.**

| Parameter | Odds Ratio | SE | 95%-CI  low | 95%-CI  high | z | p |
| --- | --- | --- | --- | --- | --- | --- |
| (Intercept) | 0.132 | 0.020 | 0.098 | 0.179 | -13.080 | <.001 |
| SES | 0.980 | 0.105 | 0.795 | 1.207 | -0.194 | .846 |
| Sex (Wom.) | **0.248** | 0.060 | 0.154 | 0.398 | -5.767 | **<.001** |
| Mig. | 0.698 | 0.153 | 0.454 | 1.073 | -1.640 | .101 |
| *Ref. Age 15* |  |  |  |  |  |  |
| Age 17 | 0.811 | 0.130 | 0.592 | 1.112 | -1.301 | .193 |
| Age 20 | **0.180** | 0.053 | 0.101 | 0.322 | -5.783 | **<.001** |
| Age 24 | **0.070** | 0.032 | 0.028 | 0.171 | -5.792 | **<.001** |

Tjur's R2 Values = 0.040

j) **Logistic Regression predicting the Topic ‘Marriage & Parenthood’ by SES, Sex, Migration Background, and Age.**

| Parameter | Odds Ratio | SE | 95%-CI  low | 95%-CI  high | z | p |
| --- | --- | --- | --- | --- | --- | --- |
| (Intercept) | 0.001 | 0.000 | 0.000 | 0.003 | -9.982 | <.001 |
| SES | **0.741** | 0.087 | 0.589 | 0.931 | -2.570 | **.010** |
| Sex (Wom.) | **2.021** | 0.476 | 1.273 | 3.207 | 2.984 | **.003** |
| Mig. | **2.267** | 0.561 | 1.395 | 3.683 | 3.305 | **.001** |
| *Ref. Age 15* |  |  |  |  |  |  |
| Age 17 | 1.683 | 1.543 | 0.279 | 10.156 | 0.567 | .570 |
| Age 20 | **7.638** | 5.847 | 1.703 | 34.243 | 2.656 | **.008** |
| Age 24 | **56.902** | 41.166 | 13.782 | 234.925 | 5.586 | **<.001** |

Tjur's R2 Values = 0.073

k) **Logistic Regression predicting the Topic ‘Celebrations & Going Out’ by SES, Sex, Migration Background, and Age.**

| Parameter | Odds Ratio | SE | 95%-CI  low | 95%-CI  high | z | p |
| --- | --- | --- | --- | --- | --- | --- |
| (Intercept) | 0.039 | 0.008 | 0.027 | 0.057 | -16.832 | <.001 |
| SES | 1.088 | 0.112 | 0.889 | 1.330 | 0.819 | .413 |
| Sex (Wom.) | 0.979 | 0.207 | 0.647 | 1.483 | -0.098 | .922 |
| Mig. | 1.228 | 0.274 | 0.794 | 1.900 | 0.922 | .356 |
| *Ref. Age 15* |  |  |  |  |  |  |
| Age 17 | 0.872 | 0.182 | 0.579 | 1.313 | -0.656 | .512 |
| Age 20 | **0.198** | 0.073 | 0.096 | 0.409 | -4.384 | **<.001** |
| Age 24 | **0.068** | 0.040 | 0.021 | 0.214 | -4.582 | **<.001** |

Tjur's R2 Values = 0.013

l) **Logistic Regression predicting the Topic ‘Driver's Licence & Vehicles’ by SES, Sex, Migration Background, and Age.**

| Parameter | Odds Ratio | SE | 95%-CI  low | 95%-CI  high | z | p |
| --- | --- | --- | --- | --- | --- | --- |
| (Intercept) | 0.001 | 0.001 | 0.000 | 0.005 | -7.173 | <.001 |
| SES | **0.467** | 0.084 | 0.328 | 0.664 | -4.241 | **<.001** |
| Sex (Wom.) | **0.394** | 0.128 | 0.209 | 0.743 | -2.874 | **<.001** |
| Mig. | 1.749 | 0.625 | 0.868 | 3.522 | 1.565 | .118 |
| *Ref. Age 15* |  |  |  |  |  |  |
| Age 17 | 6.824 | 7.401 | 0.814 | 57.182 | 1.771 | .077 |
| Age 20 | **34.272** | 34.890 | 4.660 | 252.048 | 3.472 | **.001** |
| Age 24 | **15.864** | 16.514 | 2.062 | 122.031 | 2.655 | **.008** |

Tjur's R2 Values = 0.029

# Table S4. Logistic Regression Model Outputs Including Sociodemographic Factors, Age and Internalizing Symptoms

a) **Logistic Regression predicting the Topic ‘School, Education, & Apprenticeship’ by Anxiety/Depression, SES, Sex, Migration Background, and Age.**

| Parameter | Odds Ratio | SE | 95%-CI  low | 95%-CI  high | z | p |
| --- | --- | --- | --- | --- | --- | --- |
| (Intercept) | 0.830 | 0.066 | 0.711 | 0.969 | -2.353 | .019 |
| Anx./Dep. | **0.898** | 0.032 | 0.836 | 0.964 | -2.980 | **.003** |
| SES | 0.990 | 0.037 | 0.921 | 1.064 | -0.281 | .779 |
| Sex (Wom.) | 1.044 | 0.076 | 0.905 | 1.203 | 0.590 | .556 |
| Mig. | 1.020 | 0.077 | 0.880 | 1.182 | 0.264 | .792 |
| *Ref. Age 15* |  |  |  |  |  |  |
| Age 17 | **1.339** | 0.106 | 1.148 | 1.563 | 3.706 | **<.001** |
| Age 20 | **2.423** | 0.207 | 2.049 | 2.866 | 10.341 | **<.001** |
| Age 24 | 0.862 | 0.072 | 0.731 | 1.017 | -1.764 | .078 |

Tjur’s R2 Values = 0.038

b) **Logistic Regression predicting the Topic ‘Romantic Relationships & Friendship’ by Anxiety/Depression, SES, Sex, Migration Background, and Age.**

| Parameter | Odds Ratio | SE | 95%-CI  low | 95%-CI  high | z | p |
| --- | --- | --- | --- | --- | --- | --- |
| (Intercept) | 0.205 | 0.021 | 0.168 | 0.251 | -15.442 | <.001 |
| Anx./Dep. | 1.196 | 0.056 | 1.092 | 1.310 | 3.849 | **<.001** |
| SES | 1.028 | 0.053 | 0.929 | 1.138 | 0.536 | .592 |
| Sex (Wom.) | 0.838 | 0.083 | 0.690 | 1.018 | -1.778 | .075 |
| Mig. | 0.949 | 0.097 | 0.776 | 1.160 | -0.510 | .610 |
| *Ref. Age 15* |  |  |  |  |  |  |
| Age 17 | 0.825 | 0.095 | 0.659 | 1.035 | -1.665 | .096 |
| Age 20 | 0.747 | 0.091 | 0.589 | 0.948 | -2.398 | **.016** |
| Age 24 | 1.016 | 0.118 | 0.810 | 1.275 | 0.136 | .892 |

Tjur's R2 Values = 0.006

c) **Logistic Regression predicting the Topic ‘Mental Health, Development, & Change’ by Anxiety/Depression, SES, Sex, Migration Background, and Age.**

| Parameter | Odds Ratio | SE | 95%-CI  low | 95%-CI  high | z | p |
| --- | --- | --- | --- | --- | --- | --- |
| (Intercept) | 0.090 | 0.013 | 0.069 | 0.119 | -17.340 | <.001 |
| Anx./Dep. | **1.200** | 0.070 | 1.069 | 1.346 | 3.103 | **.002** |
| SES | 1.058 | 0.065 | 0.938 | 1.193 | 0.915 | .360 |
| Sex (Wom.) | 0.816 | 0.105 | 0.633 | 1.050 | -1.582 | .114 |
| Mig. | 1.232 | 0.163 | 0.950 | 1.596 | 1.574 | .116 |
| *Ref. Age 15* |  |  |  |  |  |  |
| Age 17 | **0.713** | 0.109 | 0.527 | 0.963 | -2.205 | **.027** |
| Age 20 | 0.917 | 0.139 | 0.681 | 1.235 | -0.572 | .568 |
| Age 24 | **1.477** | 0.205 | 1.126 | 1.939 | 2.813 | **.005** |

Tjur's R2 Values = 0.009

d) **Logistic Regression predicting the Topic ‘Travelling, Vacation, & Staying Abroad’ by Anxiety/Depression, SES, Sex, Migration Background, and Age.**

| Parameter | Odds Ratio | SE | 95%-CI  low | 95%-CI  high | z | p |
| --- | --- | --- | --- | --- | --- | --- |
| (Intercept) | 0.073 | 0.011 | 0.055 | 0.097 | -18.234 | <.001 |
| Anx./Dep. | **0.835** | 0.051 | 0.741 | 0.942 | -2.944 | **.003** |
| SES | **1.434** | 0.099 | 1.252 | 1.642 | 5.209 | **<.001** |
| Sex (Wom.) | **1.588** | 0.196 | 1.246 | 2.023 | 3.743 | **<.001** |
| Mig. | 0.830 | 0.113 | 0.636 | 1.083 | -1.375 | .169 |
| *Ref. Age 15* |  |  |  |  |  |  |
| Age 17 | 1.130 | 0.159 | 0.857 | 1.489 | 0.867 | .386 |
| Age 20 | 1.086 | 0.159 | 0.815 | 1.447 | 0.561 | .574 |
| Age 24 | 0.840 | 0.133 | 0.615 | 1.146 | -1.101 | .271 |

Tjur's R2 Values = 0.019

e) **Logistic Regression predicting the Topic ‘Work & Employment’ by Anxiety/Depression, SES, Sex, Migration Background, and Age.**

| Parameter | Odds Ratio | SE | 95%-CI  low | 95%-CI  high | z | p |
| --- | --- | --- | --- | --- | --- | --- |
| (Intercept) | 0.014 | 0.004 | 0.008 | 0.024 | -15.373 | <.001 |
| Anx./Dep. | **0.810** | 0.054 | 0.712 | 0.923 | -3.175 | **.001** |
| SES | **0.790** | 0.054 | 0.691 | 0.905 | -3.418 | **.001** |
| Sex (Wom.) | 0.903 | 0.115 | 0.703 | 1.160 | -0.800 | .424 |
| Mig. | 0.978 | 0.139 | 0.740 | 1.292 | -0.157 | .875 |
| *Ref. Age 15* |  |  |  |  |  |  |
| Age 17 | **3.082** | 0.908 | 1.730 | 5.492 | 3.820 | **<.001** |
| Age 20 | **6.782** | 1.850 | 3.974 | 11.576 | 7.019 | **<.001** |
| Age 24 | **16.929** | 4.389 | 10.185 | 28.140 | 10.912 | **<.001** |

Tjur's R2 Values = 0.067

f) **Logistic Regression predicting the Topic ‘Family & family-related Events’ by Anxiety/Depression, SES, Sex, Migration Background, and Age.**

| Parameter | Odds Ratio | SE | 95%-CI  low | 95%-CI  high | z | p |
| --- | --- | --- | --- | --- | --- | --- |
| (Intercept) | 0.067 | 0.010 | 0.050 | 0.090 | -17.771 | <.001 |
| Anx./Dep. | **1.170** | 0.079 | 1.025 | 1.336 | 2.330 | **.020** |
| SES | **0.864** | 0.062 | 0.751 | 0.994 | -2.044 | **.041** |
| Sex (Wom.) | **1.573** | 0.228 | 1.184 | 2.090 | 3.127 | **.002** |
| Mig. | **1.331** | 0.194 | 1.001 | 1.770 | 1.964 | **.050** |
| *Ref. Age 15* |  |  |  |  |  |  |
| Age 17 | **0.667** | 0.101 | 0.495 | 0.898 | -2.666 | **.008** |
| Age 20 | **0.464** | 0.084 | 0.325 | 0.662 | -4.241 | **<.001** |
| Age 24 | **0.487** | 0.087 | 0.343 | 0.691 | -4.020 | **<.001** |

Tjur's R2 Values = 0.017

g) **Logistic Regression predicting the Topic ‘Moving & Living on One's Own’ by Anxiety/Depression, SES, Sex, Migration Background, and Age.**

| Parameter | Odds Ratio | SE | 95%-CI  low | 95%-CI  high | z | p |
| --- | --- | --- | --- | --- | --- | --- |
| (Intercept) | 0.023 | 0.005 | 0.015 | 0.034 | -18.009 | <.001 |
| Anx./Dep. | 1.096 | 0.073 | 0.962 | 1.249 | 1.380 | .168 |
| SES | 0.914 | 0.075 | 0.778 | 1.074 | -1.095 | .273 |
| Sex (Wom.) | **2.057** | 0.331 | 1.501 | 2.820 | 4.483 | **<.001** |
| Mig. | **0.607** | 0.109 | 0.427 | 0.862 | -2.791 | **.005** |
| *Ref. Age 15* |  |  |  |  |  |  |
| Age 17 | 0.703 | 0.187 | 0.418 | 1.183 | -1.326 | .185 |
| Age 20 | **1.714** | 0.377 | 1.114 | 2.636 | 2.450 | **.014** |
| Age 24 | **5.737** | 1.165 | 3.853 | 8.542 | 8.599 | **<.001** |

Tjur's R2 Values = 0.055

h) **Logistic Regression predicting the Topic ‘Stressful Life Events & Loss’ by Anxiety/Depression, SES, Sex, Migration Background, and Age.**

| Parameter | Odds Ratio | SE | 95%-CI  low | 95%-CI  high | z | p |
| --- | --- | --- | --- | --- | --- | --- |
| (Intercept) | 0.047 | 0.009 | 0.032 | 0.069 | -15.728 | <.001 |
| Anx./Dep. | **1.380** | 0.110 | 1.180 | 1.613 | 4.039 | **<.001** |
| SES | 1.146 | 0.112 | 0.945 | 1.389 | 1.384 | .166 |
| Sex (Wom.) | 1.275 | 0.240 | 0.882 | 1.843 | 1.292 | .196 |
| Mig. | 0.728 | 0.141 | 0.498 | 1.065 | -1.634 | .102 |
| *Ref. Age 15* |  |  |  |  |  |  |
| Age 17 | 0.679 | 0.137 | 0.457 | 1.009 | -1.914 | .056 |
| Age 20 | **0.335** | 0.092 | 0.195 | 0.574 | -3.980 | **<.001** |
| Age 24 | 1.148 | 0.223 | 0.785 | 1.679 | 0.713 | .476 |

Tjur's R2 Values = 0.015

i) **Logistic Regression predicting the Topic ‘Sports’ by Anxiety/Depression, SES, Sex, Migration Background, and Age.**

| Parameter | Odds Ratio | SE | 95%-CI  low | 95%-CI  high | z | p |
| --- | --- | --- | --- | --- | --- | --- |
| (Intercept) | 0.110 | 0.020 | 0.078 | 0.156 | -12.472 | <.001 |
| Anx./Dep. | **0.667** | 0.086 | 0.517 | 0.860 | -3.122 | **.002** |
| SES | 0.998 | 0.110 | 0.803 | 1.239 | -0.020 | .984 |
| Sex (Wom.) | **0.328** | 0.089 | 0.192 | 0.559 | -4.088 | <.001 |
| Mig. | 0.681 | 0.151 | 0.441 | 1.052 | -1.731 | .083 |
| *Ref. Age 15* |  |  |  |  |  |  |
| Age 17 | 0.798 | 0.129 | 0.581 | 1.095 | -1.399 | 0.162 |
| Age 20 | **0.177** | 0.053 | 0.099 | 0.318 | -5.809 | <.001 |
| Age 24 | **0.069** | 0.032 | 0.028 | 0.169 | -5.825 | <.001 |

Tjur's R2 Values = 0.044

j) **Logistic Regression predicting the Topic ‘Marriage & Parenthood’ by Anxiety/Depression, SES, Sex, Migration Background, and Age.**

| Parameter | Odds Ratio | SE | 95%-CI  low | 95%-CI  high | z | p |
| --- | --- | --- | --- | --- | --- | --- |
| (Intercept) | 0.001 | 0.000 | 0.000 | 0.002 | -10.008 | <.001 |
| Anx./Dep. | 0.854 | 0.101 | 0.677 | 1.078 | -1.328 | .184 |
| SES | **0.739** | 0.087 | 0.586 | 0.931 | -2.564 | **.010** |
| Sex (Wom.) | **2.242** | 0.552 | 1.384 | 3.633 | 3.279 | **.001** |
| Mig. | **2.340** | 0.584 | 1.434 | 3.816 | 3.405 | **.001** |
| *Ref. Age 15* |  |  |  |  |  |  |
| Age 17 | 1.681 | 1.542 | 0.278 | 10.150 | 0.566 | .571 |
| Age 20 | **7.639** | 5.851 | 1.702 | 34.280 | 2.655 | **.008** |
| Age 24 | **57.104** | 41.356 | 13.811 | 236.113 | 5.585 | **<.001** |

Tjur's R2 Values = 0.075

k) **Logistic Regression predicting the Topic ‘Celebrations & Going Out’ by Anxiety/Depression, SES, Sex, Migration Background, and Age.**

| Parameter | Odds Ratio | SE | 95%-CI  low | 95%-CI  high | z | p |
| --- | --- | --- | --- | --- | --- | --- |
| (Intercept) | 0.041 | 0.008 | 0.028 | 0.060 | -16.506 | <.001 |
| Anx./Dep. | 1.134 | 0.119 | 0.923 | 1.392 | 1.199 | .231 |
| SES | 1.084 | 0.111 | 0.887 | 1.325 | 0.786 | .432 |
| Sex (Wom.) | 0.886 | 0.191 | 0.580 | 1.352 | -0.562 | .574 |
| Mig. | 1.228 | 0.273 | 0.794 | 1.900 | 0.925 | .355 |
| *Ref. Age 15* |  |  |  |  |  |  |
| Age 17 | 0.875 | 0.183 | 0.581 | 1.319 | -0.637 | .524 |
| Age 20 | **0.199** | 0.073 | 0.097 | 0.410 | -4.375 | **<.001** |
| Age 24 | **0.068** | 0.040 | 0.022 | 0.215 | -4.579 | **<.001** |

Tjur's R2 Values = 0.013

l) **Logistic Regression predicting the Topic ‘Driver's License & Vehicles’ by Anxiety/Depression, SES, Sex, Migration Background, and Age.**

| Parameter | Odds Ratio | SE | 95%-CI  low | 95%-CI  high | z | p |
| --- | --- | --- | --- | --- | --- | --- |
| (Intercept) | 0.001 | 0.001 | 0.000 | 0.004 | -7.187 | <.001 |
| Anx./Dep. | 0.829 | 0.151 | 0.581 | 1.183 | -1.033 | .301 |
| SES | **0.464** | 0.085 | 0.324 | 0.664 | -4.203 | **<.001** |
| Sex (Wom.) | **0.449** | 0.159 | 0.224 | 0.898 | -2.265 | **.024** |
| Mig. | 1.768 | 0.629 | 0.881 | 3.549 | 1.603 | .109 |
| *Ref. Age 15* |  |  |  |  |  |  |
| Age 17 | 6.802 | 7.376 | 0.812 | 56.969 | 1.768 | .077 |
| Age 20 | **34.657** | 35.293 | 4.710 | 255.044 | 3.482 | **<.001** |
| Age 24 | **16.097** | 16.777 | 2.088 | 124.131 | 2.666 | **.008** |

Tjur's R2 Values = 0.030

# Table S5. Logistic Regression Model Outputs of Topic Valence by by Anxiety/Depression, SES, Sex, Migration Background, and Age.

a) **Logistic Regression predicting Negative Life Event Valence by Anxiety/Depression, SES, Sex, Migration Background, and Age.**

| Parameter | Odds Ratio | SE | 95%-CI  Low | 95%-CI  high | z | p |
| --- | --- | --- | --- | --- | --- | --- |
| (Intercept) | 0.08 | 0.01 | 0.06 | 0.11 | -16.60 | <.001 |
| Anx./Dep. | **1.52** | 0.10 | 1.34 | 1.72 | 6.44 | **<.001** |
| SES | **1.27** | 0.10 | 1.10 | 1.48 | 3.25 | **.001** |
| Sex (Wom.) | 1.07 | 0.15 | 0.81 | 1.40 | 0.48 | .631 |
| Mig. | 0.82 | 0.12 | 0.61 | 1.10 | -1.34 | .179 |
| *Ref. Age 15* |  |  |  |  |  |  |
| Age 17 | 0.82 | 0.13 | 0.60 | 1.12 | -1.23 | .220 |
| Age 20 | **0.56** | 0.10 | 0.39 | 0.80 | -3.16 | **.002** |
| Age 24 | 1.30 | 0.20 | 0.97 | 1.75 | 1.73 | .084 |

Tjur's R2 Values = 0.028

b) **Logistic Regression predicting Positive Life Event Valence by Anxiety/Depression, SES, Sex, Migration Background, and Age.**

| Parameter | Odds Ratio | SE | 95%-CI  low | 95%-CI  high | z | p |
| --- | --- | --- | --- | --- | --- | --- |
| (Intercept) | 4.09 | 0.43 | 3.32 | 5.04 | 13.29 | <.001 |
| Anx./Dep. | **0.80** | 0.04 | 0.73 | 0.88 | -4.57 | **<.001** |
| SES | **0.87** | 0.05 | 0.78 | 0.98 | -2.36 | **.018** |
| Sex (Wom.) | **0.79** | 0.08 | 0.64 | 0.98 | -2.16 | **.031** |
| Mig. | **1.32** | 0.15 | 1.05 | 1.66 | 2.40 | **.017** |
| *Ref. Age 15* |  |  |  |  |  |  |
| Age 17 | **2.01** | 0.22 | 1.63 | 2.48 | 6.46 | **<.001** |
| Age 20 | **2.96** | 0.39 | 2.29 | 3.84 | 8.22 | **<.001** |
| Age 24 | **1.37** | 0.15 | 1.10 | 1.71 | 2.82 | **.005** |

Tjur's R2 Values = 0.034
